# Distinct tau neuropathology and cellular profiles of an APOE3 Christchurch homozygote protected against autosomal dominant Alzheimer’s dementia

**DOI:** 10.1007/s00401-022-02467-8

**Published:** 2022-07-15

**Authors:** Diego Sepulveda-Falla, Justin S. Sanchez, Maria Camila Almeida, Daniela Boassa, Juliana Acosta-Uribe, Clara Vila-Castelar, Liliana Ramirez-Gomez, Ana Baena, David Aguillon, Nelson David Villalba-Moreno, Jessica Lisa Littau, Andres Villegas, Thomas G. Beach, Charles L. White, Mark Ellisman, Susanne Krasemann, Markus Glatzel, Keith A. Johnson, Reisa A. Sperling, Eric M. Reiman, Joseph F. Arboleda-Velasquez, Kenneth S. Kosik, Francisco Lopera, Yakeel T. Quiroz

**Affiliations:** 1 Molecular Neuropathology of Alzheimer’s Disease, Institute of Neuropathology, University Medical Center Hamburg-Eppendorf, Hamburg, Germany; 2 Department of Neurology, Massachusetts General Hospital, Harvard Medical School, Boston, MA USA; 3 Department of Molecular, Cellular and Developmental Biology, Neuroscience Research Institute, University of California, Santa Barbara, CA 93106 USA; 4 Center for Natural and Human Sciences, Federal University of ABC, São Bernardo do Campo, SP Brazil; 5 National Center for Microscopy and Imaging Research (NCMIR), San Diego School of Medicine (UCSD), University of California, La Jolla, San Diego, CA 92093 USA; 6 Department of Neurosciences, San Diego School of Medicine (UCSD), University of California, La Jolla, San Diego, CA 92093 USA; 7 Department of Psychiatry, Massachusetts General Hospital, Harvard Medical School, Boston, MA USA; 8 Grupo de Neurociencias de Antioquia, Facultad de Medicina, Universidad de Antioquia, Medellín, Antioquia Colombia; 9 Department of Neuropathology, Banner Sun Health Research Institute, Sun City, AZ USA; 10 Department of Pathology, Neuropathology Laboratory, University of Texas Southwestern Medical Center, Dallas, USA; 11 Institute of Neuropathology, University Medical Center Hamburg-Eppendorf, Hamburg, Germany; 12 Experimental Pathology Core Facility, University Medical Center Hamburg-Eppendorf, Hamburg, Germany; 13 Department of Radiology, Massachusetts General Hospital, Harvard Medical School, Boston, MA USA; 14 The Banner Alzheimer’s Institute, Phoenix, AZ USA; 15 Schepens Eye Research Institute of Mass Eye and Ear and Department of Ophthalmology, Harvard Medical School, Boston, MA USA

**Keywords:** Alzheimer’s disease, Dementia, Tau, PET, Transcriptomics, APOE

## Abstract

We describe in vivo follow-up PET imaging and postmortem findings from an autosomal dominant Alzheimer’s disease (ADAD) *PSEN1* E280A carrier who was also homozygous for the *APOE3* Christchurch (APOE3ch) variant and was protected against Alzheimer’s symptoms for almost three decades beyond the expected age of onset. We identified a distinct anatomical pattern of tau pathology with atypical accumulation in vivo and unusual postmortem regional distribution characterized by sparing in the frontal cortex and severe pathology in the occipital cortex. The frontal cortex and the hippocampus, less affected than the occipital cortex by tau pathology, contained Related Orphan Receptor B (RORB) positive neurons, homeostatic astrocytes and higher APOE expression. The occipital cortex, the only cortical region showing cerebral amyloid angiopathy (CAA), exhibited a distinctive chronic inflammatory microglial profile and lower APOE expression. Thus, the Christchurch variant may impact the distribution of tau pathology, modulate age at onset, severity, progression, and clinical presentation of ADAD, suggesting possible therapeutic strategies.

**Supplementary Information:**

The online version contains supplementary material available at 10.1007/s00401-022-02467-8.

## Introduction

Familial Alzheimer’s disease is characterized by its high pathological severity and early disease onset. Nevertheless, some cases belonging to a large kindred carrying the presenilin-1 (*PSEN1*) E280A mutation have shown delayed onset, suggesting possible mechanisms of disease modulation [31, 32]. Apolipoprotein E (*APOE*) haplotype variants such as *APOE4* have been associated with increased risk of sporadic Alzheimer’s disease [35]. APOE’s possible role in Alzheimer’s pathophysiology include cellular mechanisms involving neuronal and glial functions [34, 35], together with direct effects in amyloid-β (Aβ) aggregation and deposition [37]. We previously reported a *PSEN1* E280A mutation carrier from the world’s largest known autosomal dominant Alzheimer’s disease (ADAD) kindred who was spared from Alzheimer’s symptoms until her seventies, nearly three decades after the typical age of clinical onset among mutation carriers in this kindred [1]. At the time of first examination, she was found to carry two copies of the rare *APOE3* Christchurch variant (*APOE3ch*) and had severely elevated brain Aβ with limited tau pathology and neurodegeneration, as measured by in vivo PET imaging.

In vitro experiments from that report suggested that the *APOE3ch* variant may have protective effects by reducing ApoE binding to heparan sulfate proteoglycans and lipoprotein receptors involved in tau uptake and spread compared to other *APOE* variants. Since the original report, the availability of follow-up in vivo imaging and postmortem data has enabled us to further evaluate in greater detail the mechanisms of protection in this patient. In this study we aim to describe these longitudinal in vivo and postmortem findings, including an atypical regional distribution of tau pathology evident in both imaging and postmortem assessments, and gene expression profiles of neurons and neuroglia that correspond to regional vulnerability and protection against tau.

## Materials and methods

### Clinical assessments

This study was approved by the institutional review boards of the University of Antioquia, Massachusetts General Hospital, and the Schepens Eye Research Institute of Massachusetts Eye and Ear. All subjects provided informed written consent. Clinical ratings and neuropsychological tests were performed according to standard protocols as previously described [1].

### MRI and PET imaging

Structural MRI, 11C-Pittsburgh Compound B (PiB) and 18F-Flortaucipir (FTP) PET data were acquired at Massachusetts General Hospital, as previously described [29]. Imaging data from the patient was compared with data from other, typical PSEN1 E280 carriers; as well as older (> 65 years/old) sporadic AD patients described in a previous report [30]. T1-weighted structural MRI data were acquired using a Siemens 3 Tesla Tim Trio (Siemens, Erlangen, Germany; repetition time = 2300 ms; echo time = 2.95 ms; flip angle = 9°; voxel size = 1.05 × 1.05 × 1.2 mm). Images were processed with FreeSurfer (FS) version 6.0 (http://surfer.nmr.mgh.harvard.edu) to identify white and pial surfaces, standard regions-of-interest (ROI) from the Desikan atlas for PET sampling, and hippocampal volumes (HV) [5]. FS outputs were quality-checked and manually edited where necessary to ensure accurate segmentation and surface identification. Bilateral HV measures derived from FS were adjusted for intracranial volume (ICV) by regressing out the contribution of ICV on HV, using previously published parameters [26]. PiB and FTP PET were prepared and acquired according to previously published protocols [17]. All PET data were acquired on a Siemens ECAT HR + (3D mode; 63 image planes; 15.2 cm axial field of view; 5.6 mm transaxial resolution; 2.4 mm slice interval). PiB data were acquired using a 60-min dynamic protocol and analyzed by the Logan reference method with distribution volume ratio (DVR) as outcome. FTP data were acquired from 80 to 100 min post-injection in 4 × 5-min frames with the standardized uptake value ratio (SUVr) as outcome. Cerebellar gray matter was used as reference for PiB and FTP. Partial volume correction (PVC) was applied to the PET frame data using geometric transfer matrix (GTM) method for ROI analyses and an extended Muller–Gartner method (implemented in FS) for surface-based analyses [11].

PET images were affine co-registered to each subject’s contemporaneous T1 images (SPM8) and all PET data sets were sampled using FS-derived ROI. Aβ burden was represented using PiB DVR in a large, neocortical aggregate region, as well as striatum (volume-weighted average of bilateral caudate and putamen) [14]. Tau PET uptake was assessed in standard FS ROIs, as well as rhinal cortex [30] and visual cortex subregions (V1 and V2) from the FS Brodmann Area maps [8]. Change rates were expressed as annualized percent change (i.e., the difference in DVR or SUVr from baseline to follow-up, divided by the baseline value, divided by the elapsed time in years). For visualization purposes, FTP SUVr and PiB DVR images were normalized to standard (MNI) space and projected onto the fsaverage surface using FS methods (sampled at the midpoint of gray matter, surface-smoothed 8 mm).

### Neuropathological and immunohistochemical analysis

The patient died from systemic failure secondary to malignant metastatic melanoma, stage III, at 77 years of age. Brain donation took place following informed consent signature and ethical approval from the bioethics committee from the School of Medicine from the University of Antioquia. The brain presented with severe atrophy, weighing 894.3 g, and with severe atherosclerosis in all major vessels. Weight of the content of the posterior fossae: 129.5 g. Weight of cerebellum: 107.6 g. During microscopic examination we confirmed moderate cortical atrophy and presence of AD pathological hallmarks by immunohistochemistry (IHC). We examined 17 brain areas including medial frontal gyrus, superior temporal gyrus, medial temporal gyrus, inferior temporal gyrus, hippocampus, amygdala, insula, gyrus cinguli, lenticular nucleus, caudate nucleus, thalamus, inferior parietal lobule, occipital lobule, cerebellum, mesencephalon, pons, and medulla oblongata. 4 µm thick sections were stained with haematoxylin and eosin (H&E) and further processed for immunohistochemical (IHC) staining for amyloid beta (Aβ, 1:100; mouse monoclonal BAM-10, Mob410; Zytomed Systems, Berlin, Germany), hyperphosphorylated tau Ser 202 and Thr 205 (tau, 1:1500; mouse monoclonal AT8, MN1020; ThermoFisher Scientific, Dreieich, Germany), Ionized calcium-binding adapter molecule 1 (Iba1, 1:500; rabbit polyclonal, 019-19741; FUJIFILM Wako chemicals GmbH, Neuss, Germany), Transmembrane Protein 119 (TMEM119, 1:100; rabbit polyclonal, 400 102; Synaptic Systems, Göttingen, Germany), Cluster of Differentiation 68 (CD68, 1:100; rat monoclonal, HS-460 017; Synaptic Systems, Göttingen, Germany), Glial Fibrillary Acidic Protein (GFAP, 1:200; mouse monoclonal M0761, DAKO GmbH, Jena, Germany), Nuclear receptor RZR-β (RORB, 1:500; rabbit polyclonal, HPA008393; Merck Millipore, Darmstadt, Germany) and Apolipoprotein E (ApoE, 1:100; Goat polyclonal, AB947, Merck Millipore, Darmstadt, Germany). Automatic immunostaining was performed with a Ventana Benchmark XT system (Roche AG, Basel, Switzerland) according to manufacturer instructions. Briefly, after dewaxing and inactivation of endogenous peroxidases (PBS/3% hydrogen peroxide), antibody specific antigen retrieval was performed, sections were blocked and afterwards incubated with the primary antibody. For detection of specific binding, the Ultra View Universal 3,3´-Diaminobenzidine (DAB) Detection Kit (Ventana, Roche) was used which contains secondary antibodies, DAB stain and counter staining reagent. Sections were scanned using a Hamamatsu NanoZoomer automatic digital slide scanner (Hamamatsu Photonics, Hamamatsu, Japan) and obtained images of whole stained sections at a resolution of at least 1 pixel per µm. Signal of total area and signal integrated density were assessed using ImageJ Software (version 1.52p, NIH, Bethesda, MA, USA.) in the brown (DAB) color channel after performing color deconvolution and thresholding. Data was analyzed using GraphPad Prism 6 (GraphPad Software, Inc., La Jolla, CA, USA) and R statistical software (R Foundation for Statistical Computing, Vienna, Austria). Analyses, including distribution analysis and correlation analysis, were performed using Spearman’s *ρ* test. Brain color maps were created using the cerebroViz package for R [3] for a general reference of the anatomical distribution of pathology and IHC signal. Statistical significance of all analyses was determined with *p ≤ 0.05, **p ≤ 0.01 and ***p ≤ 0.001.

### Electron microscopy of human specimen

Surgically excised specimens were immediately placed in 4% paraformaldehyde and 2.5% glutaraldehyde in PBS pH 7.4 and kept in the fridge until shipment to UCSD. Upon arrival, specimens were transferred to a freshly prepared ice-cold solution of 4% paraformaldehyde and 2.5% glutaraldehyde in 0.15 M cacodylate buffer pH 7.4 containing 2 mM CaCl_2_ and kept overnight in the fridge. The next day, the samples were sectioned using a vibratome (Leica) to 100 micron-thick sections and placed in the same fixative solution overnight in the fridge. Slices were then washed 3 times in 0.15 M cacodylate buffer pH 7.4 containing 2 mM CaCl_2_, and post-fixed in 2% OsO_4_/1.5% K_4_Fe(CN)_6_ (Sigma-Aldrich, St. Louis, MO) for 1 h at room temperature (RT) in 0.15 M cacodylate buffer. After 3 washes with double distilled water (ddH_2_O), 5 min each at RT, slices were placed in filtered 1% thiocarbohydrazide solution for 30 min at RT, rinsed again in ddH_2_O (3 times, 5 min each) and then placed in 2% OsO_4_ for 1 h at RT. After this second osmium step, the sections were rinsed at RT in ddH_2_O (3 times, 5 min each) and left in filtered 2% uranyl acetate aqueous solution overnight at 4 °C. The next day, after three washes in ddH_2_O, 5 min each, at RT, en bloc Walton’s lead aspartate staining was performed for 30 min at 60 °C. Following three washes for 5 min each in ddH_2_O at RT, sections were dehydrated using a series of ice-cold graded ethanol solutions for 10 min each, and an additional 100% acetone step at RT for 10 min. Slices were infiltrated with a solution of 50% acetone: 50% Durcupan ACM epoxy resin (Electron Microscopy Sciences) overnight and then placed into fresh 100% Durcupan for three additional days. Lastly, the sections were embedded using two mold-release coated glass slides and kept at 60 °C for 72 h. X-ray microCT imaging was performed using Xradia 510 Versa (Carl Zeiss Microscopy) to evaluate tissue preservation and homogeneity before proceeding to EM imaging. Ultra-thin sections were cut using a diamond knife (Diatome) at a thickness of 70–90 nm. Thin sections were examined using a FEI Technai 12 Spirit (Thermo Fisher Scientific) transmission electron microscope operated at 80 kV. Micrographs were produced using a Tietz 2 k by 2 k CCD camera and collected using the SerialEM package. Images were then processed and analyzed using Fiji software (a bundled version of ImageJ, see above).

### Single nuclei RNA sequencing

#### Nuclei isolation

Hippocampal formation (HIP), frontal cortex (middle frontal gyrus, Brodmann area 46; FC) or occipital cortex (Brodmann areas 19 and 18, OL) were dissociated, and nuclei isolation was performed separately for each of these regions using the Nuclei Isolation Kit: Nuclei EZ Prep (Sigma, #NUC101) as described by Habib et al. [13]. Briefly, tissue samples were Dounce homogenized in 2 ml of ice-cold EZ PREP and incubated on ice for 5 min. Following initial Dounce homogenization, an additional 2 ml of EZ PREP was added and the samples were incubated for 5 min. Nuclei suspension was centrifuged (500×*g*, 5 min and 4 °C) washed 1X in ice-cold EZ PREP buffer, and 1X in Nuclei Suspension Buffer (NSB; consisting of 1X PBS, 1% (w/v) BSA and 0.2 U/μl RNase inhibitor (Clontech, #2313A), resuspended in 1 ml of NSB and filtered through a 40 μm cell strainer. Nuclei were stained with SYTOX green (1:1000) and counted twice. A final concentration of 1000 nuclei per µl was used for loading onto the 10X Chromium (10X Genomics). Library construction was performed using the Chromium Single Cell 3′ Library and Gel Bead Kit v3.1 (10X Genomics) and sequencing on one high-output lane of the NextSeq 4000 (Illumina).

#### Mapping single nuclei reads to the genome

Using the Grch38 (1.2.0) reference from 10 × Genomics, we made a pre-mRNA reference according to the steps detailed by 10X Genomics (https://support.10xgenomics.com/single-cell-gene512expression/software/pipelines/latest/advanced/references). Sequencing reads were aligned to the human pre-mRNA reference transcriptome using the 10 × Genomics CellRanger pipeline (version 3.0.0; RRID: SCR_017344) with default parameters.

#### Quality control for expression matrix

Downstream analysis was performed using Seurat 4.0 in RStudio Version 4.1.0. An individual Seurat object was generated for each sample. Cells with fewer than 200 detected genes and with more than 5% of reads mapped to mitochondrial genes were filtered out. Doublets were identified using the DoubletFinder package [24] and removed assuming a doublet rate formation of 3%.

#### Data processing, analyses, visualization, and differential expression testing

The samples were then merged into a single Seurat object and SAVER (version 1.1.2) was used for missing data imputation [15]. Data were then normalized and scaled by using the Sctransform function in Seurat using the default parameters. Anchor-based sample Integration was performed on the normalized counts, with the number of features in the anchor finding process set to 3000. Non-linear dimensionality reduction was performed by running UMAP on the first 20 PCs. Clustering was performed on the top 20 PCs as input in the FindNeighbors function, and a resolution of 0.2 in the FindClusters function, which resulted in 12 clusters and was in good agreement with the expression of known marker genes for cell types found in human brain. To enable an unbiased verification of the cluster identities, the top marker genes per cluster were computationally determined with the FindAllMarkers function, using the Wilcoxon rank sum test with a FDR-corrected *p* value ≤ 0.01. Only positive marker genes were considered, and these markers were used with known cell-type markers from the literature [12, 19, 20, 23] to carefully assign cluster identities.

#### Identification of differentially expressed genes in cell-type subpopulations

After cell-type annotation, for differential gene expression analysis across regions, each cell type was subseted, re-clustered and differential gene expression analysis was performed. To identify genes differentially expressed by a cell-type subpopulation across different regions (i.e., FC, HIP or OL), we performed differential expression based on the non-parametric Wilcoxon rank sum test accessed through the FindMarkers function in Seurat. We compared the differences between OL vs HIP, OL vs FC and HIP vs FC for each cell subtype. An FDR-corrected *p* value of ≤ 0.01 and a logFC threshold = 0.5 was used. For heatmaps of relative gene expression across cell-type subpopulations or across brain regions, SCT normalized counts of each gene were *z*-score transformed across all cells and then averaged across cells in each cluster to enhance visualization of differences among clusters. Thus, genes with high relative expression had above-average expression (positive *z*-scores) and genes with low relative expression had below-average expression (negative *z*-scores).

#### Correlation analysis

For all three regions we ran a correlation analysis between average expression of SCT normalized values of APOE and all other genes expressed in microglia and astrocytes, which were the two cell types that showed expression of APOE. We performed Gene ontology enrichment analysis on the list of genes with a statistically significant (*p* ≤ 0.05) r correlation coefficient ≥  ± 0.5 using PANTHER Gene List Analysis tools and Clusterprofiler [38].

#### Targeted analysis of microglia

Differentially expressed genes (DEG) identified in FC, HIP and OL were contrasted with gene signatures identified for acute (using LPS), and neurodegenerative chronic responses in a transgenic AD murine model as described by Krasemann et al. [18].

## Results

### Neuroimaging findings

The patient’s cognitive profile at age 72 showed deficits in memory recall, with relatively preserved verbal learning and recognition, naming, verbal fluency, and visuospatial skills. She was then diagnosed with mild cognitive impairment, hypertension, and mixed dyslipidemia, and received statin treatment. A 24-months follow-up assessment showed relatively stable cognitive function, but increased need for assistance in all instrumental activities of daily living (ADLs) and some basic ADLs mostly due to limited mobility. Her neurological examination was significant for frontal release signs. She was diagnosed with mild dementia at age 75, and her last assessment, completed at age 76, revealed further decline in cognition across all domains and ADLs (Supp. Table 1, online resource). Her cognitive profile then was indicative of global deficits, consistent with the atrophy noted in the postmortem neuropathological findings. Nine months before her death, she developed multiple hyperpigmented nodular lesions on her face and abdomen, associated with neck, torso, and inguinal soft tissue lesions. She was diagnosed with metastatic melanoma with multiorgan involvement. The family opted for palliative measures, and she died at home at age 77.

**Fig. 1:**
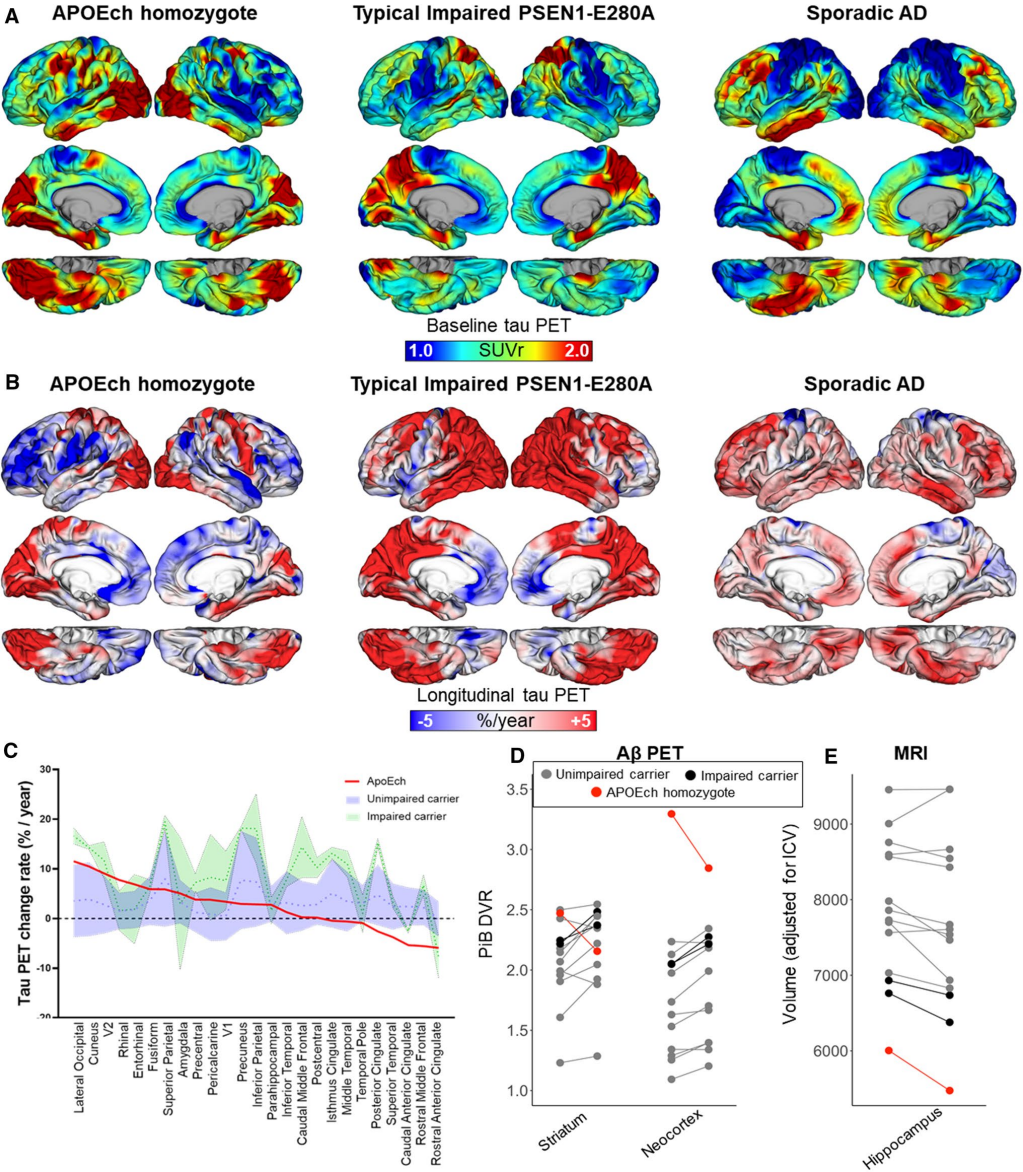
Longitudinal tau PET imaging measures in an APOE3ch homozygote. **A** Surface rendering of tau PET (Flortaucipir) images (standardized uptake value ratio, SUVr), at baseline, 3-year follow-up (center), and **B** rate of change (expressed as %/year), in the APOE3ch homozygote, (left) a typical PSEN1-E280A impaired carrier (center) and a sporadic AD case (right). **C** Distribution area plot showing annualized percent change rates in tau PET for APOE3ch homozygote (red line) relative to unimpaired (blue) and impaired (green) PSEN1-E280A carriers. Regions along the x-axis are ordered from highest to lowest change rate observed in the APOE3ch homozygote. **D** Spaghetti plots of Aβ PET (Pittsburgh Compound B, PiB) measurements at baseline and 2-year follow-up, **E** Structural MRI measurements at baseline and 2-year follow-up of hippocampal volume

As part of the COLBOS longitudinal biomarker study [31], she underwent in vivo MRI and positron emission tomography (PET) measurements at age 73 and 75. At baseline, she had very high PET measurements of Aβ plaque burden, particularly in neocortex, consistent with the overproduction of Aβ previously observed in *PSEN1* E280A carriers, drawn out over nearly four decades [9, 31]. Aβ plaque burden appeared to decrease during the three-year follow-up period (Fig. 1D) but remained at higher levels compared to the other *PSEN1* E280A carriers. Tau PET revealed an anatomical pattern of accumulation that deviated from that seen in sporadic Alzheimer’s disease (AD) as well as that observed in other impaired *PSEN1* E280A carriers, both cross-sectionally and longitudinally (Fig. 1A, B). In particular, tau PET showed relative sparing of temporal and parietal neocortex with abnormally elevated tau burden in the occipital cortex. Marked increases in PET measurements of tau tangle burden were observed during the three-year follow-up period, including in medial temporal regions (entorhinal cortex: 6.9%/year, amygdala: 5.1%/year) and most prominently in occipital cortex (lateral occipital: 11.5%/year) (Fig. 1C) consistent with her cognitive and functional decline during this period. On structural MRI measurements, her brain showed greater global atrophy relative to younger impaired *PSEN1* mutation carriers (see Fig. 1E, consistent with autopsy findings). All these findings indicated a distinctive disease progression pattern.

### Neuropathological findings

**Fig. 2:**
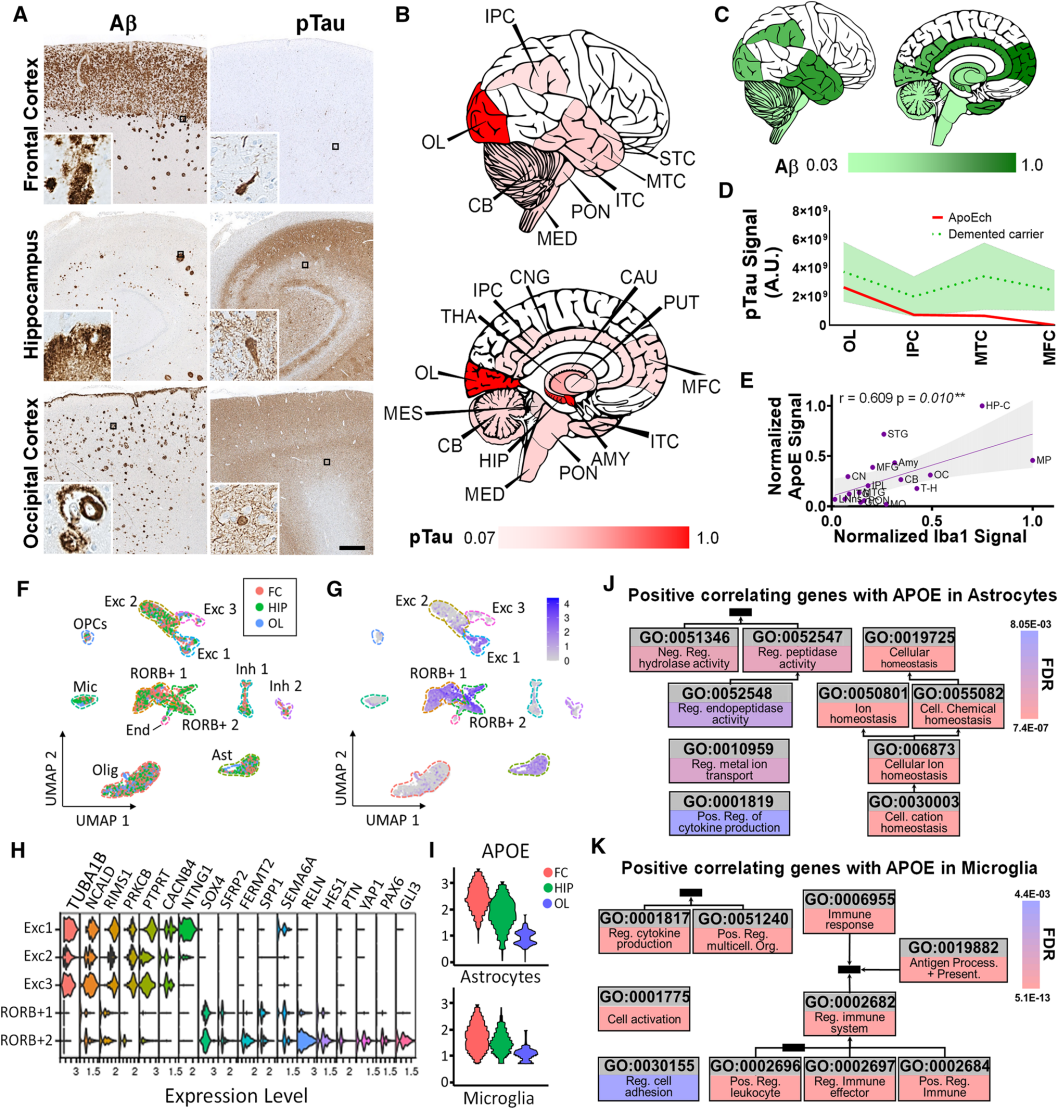
Neuropathological and molecular characterization of an ADAD PSEN1E280A mutation carrier with two copies of the APOE3ch variant. **A** Representative panels for tau and Aβ pathology in frontal cortex, hippocampus, and occipital cortex. Insets show specific pathological features found in each brain area, such as NFT, dystrophic neurites and diffuse tau pathology, together with diffuse, core Aβ plaques and CAA. Bar = 500 µm. **B** Graphic representation of general distribution and intensity of tau pathology signal with normalized lower and maximum values represented in red intensity. *MFC* medial frontal cortex, *STC* superior temporal cortex, *MTC* middle temporal cortex, *ITC* inferior temporal cortex, *HIP* hippocampus, *AMY* amygdala, *CNG* cingulate cortex, *PUT* putamen, *CAU* caudate, *THA* thalamus, *IPC* inferior parietal cortex, *OL* occipital lobe, *CB* cerebellum, *MES* mesencephalon, *PON* pons, *MED* medulla oblongata. **C** Graphic representation of distribution and intensity of Aβ signal with normalized lower and maximum values represented in green intensity. **D** Distribution area plot showing tau integrated density signal in cortical areas in APOE3ch homozygote (red line) relative to impaired (green) PSEN1-E280A carriers. Areas are ordered according to the highest to lowest tau integrated density in the APOE3ch homozygote. **E** Correlation scatter plot for ApoE signal intensity against Iba1 signal intensity in all areas studied in **B**. A positive correlation was identified as statistically significant. (**p ≤ 0.01). **F** UMAP clustering plot of snRNA sequencing data from the frontal cortex (FC, red dots), hippocampus (HIP, green dots) and occipital cortex (OL, blue dots). Identified clusters include excitatory neurons (Exc 1, 2 and 3), oligodendrocyte precursor cells (OPCs), microglia (Mic), RORB positive neurons (RORB + 1 and 2), inhibitory neurons (Inh 1 and 2), endothelial cells (End), oligodendrocytes (Olig) and astrocytes (Ast). **G** UMAP clustering plot of snRNA sequencing data from the analyzed areas depicting RORB expression levels in the different clusters. **H** Violin plots for differential expression of representative genes between excitatory neurons and RORB + neurons clusters. Excitatory clusters differentially express functional synaptic genes while RORB1 clusters express neuronal development genes. **I** Violin plots for APOE expression in FC, HIP and OL in astrocytes and microglia. **J** Top gene ontology terms from overrepresented genes positively correlating with APOE expression in astrocytes from frontal cortex, hippocampus and occipital cortex. **K** Top gene ontology terms from overrepresented genes positively correlating with APOE expression in microglia from frontal cortex, hippocampus and occipital cortex

The family donated her brain for research in accordance with ethical guidelines. The brain was collected with a postmortem delay of 200 min, it presented with global atrophy, weighing 894.3 g, and with severe atherosclerosis in all major vessels. Microscopic examination confirmed moderate thinning of the gray matter ribbon and presence of AD pathological hallmarks by immunohistochemistry (IHC). We examined 17 brain regions to assess Aβ and tau pathology distribution. The case was classified as a Braak VI due to extensive pTau isocortical pathology, Thal V due to the severity and wide distribution of Aβ pathology, and as CERAD C given the age of the patient and the high frequency of Aβ neuritic plaques. Furthermore, the integration of these pathological assessments following the NIAA guidelines gives a classification of AD neuropathological changes of A3B3C3 [16], that can be interpreted as high, similar to other PSEN1E280A cases. Although the case was classified as Braak VI [4], the distribution was atypical, with the highest density of tau signal in the hippocampus, amygdala and occipital cortex (Brodmann areas 17 and 18) and an unusually low density of pathological tau in other typically-involved structures, including negligible tau signal in the frontal cortex (Fig. 2A, B, Supp. Figure 1, online resource). In contrast, Aβ pathology distribution showed an expected [6, 32] fronto-temporal predominant pattern (Fig. 2A–C, Supp. Figure 2, online resource). These postmortem findings are therefore similar to our in vivo tau PET findings, confirming the presence of a distinctive anatomical pattern when compared with other ADAD cases (Fig. 2C). The case was screened for both, TDP-43 and alpha synuclein pathologies. There were some granular diffuse TDP-43 intraneuronal deposits in the hippocampus and the amygdala, while there was no evidence of synucleopathy in any of the regions studied (Supp. Figure 3, online resource).

Tau pathology presented across isocortical regions (including temporal, parietal, and occipital cortex) with neuropil threads (NPTs) as well as dystrophic neurites (DNs), evenly distributed between supra and infragranular layers. In somatosensory cortices, namely the superior temporal gyrus, inferior parietal lobule, and striate cortex, NPTs mostly compromised layer II, the upper part of layer III, and layer V. Neurofibrillary tangles (NFTs) in the cortex, including the insula, showed no particular layering pattern except for the cingulate gyrus, where they were restricted to layer V. Among subcortical structures (including hypothalamus, caudate, and lenticular nucleus), NPTs were homogenously distributed without visible NFT formation. In sections of the midbrain, pons, and medulla oblongata, tau immunoreactivity was low with a predominance of NPTs. In the midbrain, there was a cluster of mature NFTs restricted to the borders between the tectum and the tegmentum whereas in the medulla oblongata there was pre-tangle formation in the inferior olivary complex. Finally, in the hippocampus, NFTs were distributed across Amon’s horn with lower density in the CA3 region, and abundant dystrophic neurites in CA1. Tau immunoreactivity in the subiculum comprised mainly NPT without NFT and was noticeably low in the pre-subiculum. Scarce tau deposits were seen in frontal cortex and no tau immunoreactivity was detected in the meninges or the cerebellum, neither perivascular nor subpial deposition (Fig. 2A, Supp. Table 1, and Supp. Figure 1, online resources). Cortical differences for tau deposits were confirmed by ultrastructural analysis (Supp. Figure 4, online resource). We did not see evidence of aging-related tau astrogliopathy or argyrophilic grain disease in the studied areas.

On the other hand, Aβ pathology was variable amongst studied areas, leading to a Thal phase 5 classification [33] (Supp. Figure 5A, online resource), with diverse severity and Aβ plaque subtypes. Cortical structures presented with varied subtypes and distribution of Aβ plaques. The different hippocampal subfields varied in the type of plaques. In CA3, there were predominantly cored plaques with scant neuritic plaques, while CA2 lacked Aβ burden. Cored plaques as well as large diffuse plaques were observed in CA1, and mainly small diffuse plaques were visible in the subiculum. In the pre-subiculum, there was a profuse accumulation of Aβ in the form of diffuse plaque through the cerebral cortex without a particular layer distribution. In the entorhinal cortex, there were diffuse plaques in the subpial and layer I segments, whereas from layer II through VI, there were cored as well as neuritic plaques (Fig. 2C, Supp. Table 2, and Supp. Figure 2, online resources). Interestingly, the occipital cortex had mainly neuritic and cored plaques, and it was the only neocortical structure that showed Aβ deposits in blood vessels (cerebral amyloid angiopathy, CAA) (Fig. 2C, Supp. Figure 5B, C, online resource), while the cerebellum was the only area with CAA in leptomeningeal vessels (Supp. Figure 5B, C, online resource), which contrasted to more generalized distribution of CAA in other *PSEN1* E280A cases [7, 22]. Intraparenchymal CAA density correlated positively with tau IHC signal intensity, with amygdala and occipital cortex showing both CAA and high signal intensity for tau (Supp. Figure 5D, online resource). Notably, there was no correlation between Aβ burden and CAA density (Supp. Figure 5E, online resource). We have recently reported mild to severe small vessel disease findings in the *PSEN1* E280A population, together with enlargement of perivascular spaces and decreased perivascular astrocytic podocytes [22]. The APOE3ch case presented with some of these features, including cortical microinfarcts, arteriosclerosis and perivascular space enlargement. Some of these features were found to be above the average identified in this population, possibly related to the older age at the time of death (Supp. Figure 6, online resource).

ApoE, Iba1 (microglia) and GFAP (astrocyte) signal and distribution were also evaluated in all regions to investigate any relationship between ApoE presence and glial reactivity to pathology (Supp. Figure 7A–C, online resource). ApoE signal showed a plaque-like pattern with stronger intensity in the areas more affected by tau pathology. Iba1 signal showed larger and more branched microglia in the hippocampal and occipital cortex, and ApoE and Iba1 immunoreactivity were positively correlated (Fig. 2E). Regarding microglia profiles, cortical Iba1 signal was higher in occipital cortex when compared to frontal and temporal cortices, while TMEM119 signal was higher in frontal cortex and CD68 signal was higher in occipital cortex. CD68/TMEM119 signal ratio was higher in occipital cortex as well, indicating higher activation of microglia in this area (Supp. Figure 8, online resource). GFAP staining detected strong astrocytic immunoreactivity that was negatively correlated with the Aβ signal (Supp. Figures 7C, 9, online resource). Finally, the distribution pattern of pathological tau was not correlated with those of Aβ, ApoE, microglial marker Iba1 and the astrocytic marker GFAP (Supp. Figure 9, online resource). In summary, there were distinctive regional and local tau pathology and glial reactivity profiles suggestive of protection in most of the studied areas, except for areas also presenting with parenchymal CAA.

### Single nuclei transcriptome findings

For a deeper understanding of regional pathological differences observed in this patient, we performed single nuclei RNA sequencing (snRNA seq) in frontal cortex, hippocampus and occipital cortex (Supp. Table 4, Supp. Figure 10, online resources). Clustering of these data revealed well-defined populations of both excitatory and inhibitory neuronal populations as well as glial populations (Supp. Tables 5, 6, online resource). Remarkably, a subset of excitatory neurons was clustered independently in frontal cortex and hippocampus, characterized by high expression of RORB (Fig. 2F, G, Supp. Figure 11A, B, online resource). Recently, RORB positive excitatory neurons were identified as being more abundant in the entorhinal cortex of non-demented cases unaffected by tau pathology in this region [21]. In our case, RORB positive neurons showed lower expression of synaptic-related genes while showing higher expression of genes associated with neurodevelopment (GO: 0030154, GO:0048699 and GO:0050767) (Fig. 2H, Supp. Figure 11C, online resource). By comparing expression levels and cluster distribution of RORB positive neurons in previously published datasets (Syn21788402, GSE97930) [20, 21], we identified that RORB showed higher expression and a more distinctive cluster distribution (Supp. Figure 12, online resource). Furthermore, we compared the expression profile of neurodevelopmental-associated genes between the RORB positive neurons identified in the APOE3ch case and previously published datasets. Even though neurodevelopmental genes or genes associated with neuronal morphogenesis are presented in RORB positive neurons in all datasets (ours and previously published controls), the specific set of genes were different (Supp. Figure 13, online resource). The identification of RORB positive neurons in the areas less affected by tau, together with their distinctive expression profile, supports a selective response of vulnerable neuronal populations in this case. Regarding other cellular populations, we identified differential gene expression profiles in oligodendrocytes, astrocytes, and microglia, with specific subsets of genes highly expressed in the frontal cortex while being downregulated in the occipital cortex for the same cell types (Supp. Figures 14A–16 and 18, online resources). *APOE* was among the differentially expressed genes in astrocytes and microglia with an expression gradient from high in frontal cortex down to low in occipital cortex (Fig. 2I, Supp. Figure 14B, C, online resource).

We explored which genes followed the same expression gradient as *APOE* by identifying gene expression correlates in astrocytes (Supp. Figure 17A, online resource) and microglia (Supp. Figure 18A, online resource). Our analyses revealed a positive correlation for a subset of homeostatic genes in astrocytes (Fig. 2J, Supp. Figure 17B, Supp. Table 7, online resources), with stronger correlations in frontal cortex and hippocampus (Supp. Figure 18C, online resource). On the other hand, we determined a positive correlation for immune response regulation in microglia (Fig. 2K, Supp. Figure 19B, Supp. Table 8, online resources), with stronger correlations in occipital cortex (Supp. Figure 19C, online resource). Given the characteristic morphology of microglia found in the occipital cortex and increased correlation of immune response gene expression, we performed gene expression targeted analysis in microglia, taking as reference neurodegenerative microglia signatures for chronic (Supp. Figure 20A, online resource) and acute (Supp. Figure 21A, online resource) responses, according to Krasemann et al. [18]. We identified a subset of genes for cellular response in hippocampus and cellular locomotion in occipital cortex, indicating active inflammatory processes, while only some genes related to reactive oxygen species (ROS) response were found in frontal cortex (Supp. Figure 20B, online resource). In addition, only frontal cortex showed a clear signature for acute immune response, with a subset of genes overrepresented for cellular energy regulation (Supp. Figure 21B, online resource). All these findings together suggest that astrocyte homeostasis is responsive to *APOE3ch* expression levels in more protected brain areas, while lower levels of *APOE3ch* expression can be associated with a more deleterious, chronic microglial response.

## Discussion

Our current findings extend several observations suggested in the original report about this patient who was a carrier of the autosomal dominant Alzheimer’s disease *PSEN1* E280A variant and homozygote for the APOE3 Christchurch variant, and who only developed cognitive impairment thirty years after the typical age of clinical onset for her kindred [1]. For instance, amyloid and tau pathologies were differentially impacted in a pattern not observed in other forms of AD. Longitudinal follow-up measures of in vivo PET imaging and postmortem neuropathology analyses confirmed this observation. This discrepancy between Aβ and tau pathology indicates that putative *APOE3ch* protective molecular mechanisms may be modulated further by other pathological events during the course and progression of disease. We have recently reported a prominent role for tau pathophysiology in the modulation of age of onset in this ADAD population [32]. Similar to our current findings, AD protected cases showed decreased tau cortical pathology, with the exception of the occipital cortex which showed the same degree of tau pathology regardless of age of onset [32]. Another finding supporting the distinct features of AD pathology in this patient is the almost exclusive presence of CAA in the occipital cortex. This finding is consistent with previous reports in sporadic AD cases of increased CAA severity in this region [2], possibly related to Aβ drainage and structural characteristics of the brain vascular system. Moreover, an association between parenchymal CAA pathology, perivascular tau pathology [36], and neuronal tau pathology severity has been described [27], consistent with this case’s unique pattern of pathology in which the few regions affected with CAA showed also higher tau pathology. We suggest that, even though the homozygous *APOE3ch* variant likely rendered general protection for tau pathology in this patient, it could not render specific protection against CAA-related tau pathology in the occipital cortex, possibly due to the local effects of CAA pathology and associated tau deposition. Additionally, pathological tau has been reported in the occipital cortex of aged non-demented subjects [28]. Given the advanced age of the *PSEN1* E280A *APOE3ch* case, the pathology pattern observed in this patient can be a cumulative result of different processes including senescence.

Of note, even though neuroimaging and neuropathological findings revealed severe pathology in the occipital cortex, a region affected in Posterior Cortical Atrophy (PCA) [5], this patient did not present clinical features consistent with a PCA syndrome, such as visual and/or spatial impairments. Further, in PCA the neurodegenerative process is known to follow an occipito-parietal to temporal lobe progression, and in the E280A APOE3ch case we observed a relative sparing of parietal and frontal cortices (Supp. Fig. 22, online resource), which may explain, at least in part, the preserved visuo-spatial functioning in this case.

The specific gene expression profile presented by RORB positive neurons in frontal cortex and hippocampus provides further evidence for complex mechanisms of protection in this patient. The expression of neurogenesis-related genes in these neurons can be contrasted to previous findings in control cases [20, 21] and decreased adult neurogenesis in AD patients [25]. We discarded the possibility that the expression of proliferative genes in these cells would indicate neural stem cell activity or that they could be related to the metastatic melanoma present in the patient (Supp. Figure 23, online resource), suggesting alternative functions of neurogenesis-related genes in this context. It is known that ApoE has a relevant role in AD pathophysiology in astrocytes and immune response to the disease [35, 36]. Depletion of astrocytic *APOE4* reduces tau levels [35], while ApoE specific immunotherapy against human ApoE reduces CAA [37], both findings obtained in murine models. ApoE also functions as an activator of microglia response against Aβ plaque deposition [34]. In the *APOE3ch* case, a regional decreasing gradient of *APOE* expression levels from frontal cortex to occipital cortex was associated with astrocyte homeostasis and the presence of activated neurodegenerative microglia in occipital cortex, the most affected area with lower *APOE* expression levels, supporting the idea of a dose-dependent mechanism of protection with the participation of both cell types. It is intriguing that, in contrast to *APOE* expression levels, ApoE IHC signal intensity did not follow a gradient pattern but was more easily associated with AD pathology regional severity. This might be explained by pathological ApoE protein aggregation, together with or independent to other protein aggregates as it has been observed in demented cases [10]. It should be also noted that in general, gene expression does not necessarily represent actual protein levels and further studies would be necessary to confirm our findings regarding the pathways involved.

In summary, we described the in vivo follow-up and postmortem findings of an ADAD *PSEN1* E280A carrier who also was found to be homozygous for the *APOE3* Christchurch variant, and who was protected against AD symptoms for almost three decades. We identified a distinct anatomical pattern of tau pathology, including atypical accumulation of in vivo tau pathology, as measured by PET imaging, and an unusual regional distribution of tau aggregates and CAA pathology. Our findings suggest that *APOE3ch* may have a regionally specific role in modifying the effect of tau on the severity, progression, and clinical presentation of AD, and that its protective role is associated with its expression and resulting impact on astrocytic and microglia function. We previously reported the impact of tau pathology as a disease onset modifier [32] and disease progression marker in vivo [31] in this ADAD population. This study has some limitations, the most prominent being that the findings are based on the analyses of one case and the fact that mere associations cannot address causal connections by themselves. Nevertheless, the findings draw a remarkable picture on the relationship between ApoE function as a modulator of pathology in Alzheimer’s disease. Further studies involving more protected patients would be ideal to compare and validate our findings, but locating another ADAD case homozygous for the *ApoE3ch* variant might be very challenging.

Our findings provide evidence supporting tau pathology as the main driver of cognitive decline in *PSEN* E280A ADAD, as suggested by other previous findings in this population [31, 32], and that the protection against this pathology should be clinically effective. The strong putative protective effect of homozygous *APOE3ch* in this patient suggests that ApoE may be one of the main modulators of tau pathology in AD, possibly via different mechanisms including aggregation by modifying ApoE heparin binding [1], and homeostatic regulation of glial cells. More studies are necessary to further examine the interactions between ApoE and tau, to clarify the role of adult neurogenesis in AD protection, and to advance possible therapies mimicking the effect of *APOE3ch* in AD pathophysiology.

## Supplementary Information

Below is the link to the electronic supplementary material.

Supplementary file 1 (PDF 5464 KB)

Supplementary file 2 (XLSX 300 KB)

Raw sequencing data have been deposited at NCBI Gene Expression Omnibus (GEO) and are accessible through Accession Number GSE206744.
