# Supplementary material for: Distinct tau neuropathology and cellular profiles of an APOE3 Christchurch homozygote protected against autosomal dominant Alzheimer’s dementia
Source: Acta Neuropathol. 2022 Jul 15;144(3):589–601. doi: 10.1007/s00401-022-02467-8 (PMC9381462; doi:10.1007/s00401-022-02467-8)
Supplement: Supplementary file 1 — (PDF 5464 KB) [file 401_2022_2467_MOESM1_ESM.pdf]

Supplementary Table 1. Test Scores and Percentiles adjusted for age & education.

Cognitive Test	Initial Visit	12-Mo Visit	24-Mo Visit	36-Mo Visit	Mean (SD)**
	Raw Scores (Percentiles**)				
**MMSE/30***	18 (1st)	16 (<1st)	19 (4th)	16 (<1st)	22 (1.7)
**Naming/15**	9 (7th)	9 (7th)	8 (3rd)	8 (3rd)	12.03 (2.1)
**CERAD Word List Learning/30**	10 (5th)	6 (<1st)	8 (1st)	8 (1st)	15.64 (3.44)
**CERAD Word List Delayed Recall/10**	0 (<1st)	0 (<1st)	0 (<1st)	0 (<1st)	5.77 (1.98)
**CERAD Word List Recognition/10**	9 (27th)	7 (<1st)	4 (<1st)	1 (<1st)	9.58 (0.98)
**CERAD Praxis- Copy/11**	8 (18th)	9 (39th)	6 (1st)	3 (<1st)	9.42 (1.57)
**CERAD Praxis-Recall/11**	0 (<1st)	2 (3rd)	0 (<1st)	0 (<1st)	7.52 (2.84)
**Semantic Fluency (Animals)**	12 (13th)	12 (13th)	12 (13th)	10 (5th)	16.97 (4.3)
**Phonemic Fluency ("F" Words)**	10 (13th)	11 (14th)	10 (13th)	5 (5th)	23.75 (11.89)
**Raven's Matrices Test/12**	7 (27th)	7 (27th)	8 (47th)	6 (14th)	8.21 (2.13)
**Rey-Osterrieth Complex Figure**	7 (<1st)	23 (19th)	21.5 (10th)	6.5 (<1st)	26.76 (4.18)
**GDS/15**	9	2	9	2	
**EDG/7**	3	3	4	5	

Note. Abbreviations: MMSE, Mini-Mental State Examination; CERAD, Consortium to Establish a Registry for Alzheimer's Disease Test Battery; GDS, Geriatric Depression Scale; EDG, Global Deterioration Scale.

* MMSE subtests that require reading and writing skills were not administered due to her limited literacy skills. Her maximum possible score was 23 (instead of 30).
** Percentiles were calculated using norms for this Colombian population[1]. Percentiles between 25 and 75 place her performance in the average range for her age and education. Percentiles between 9 and 25 classify her performance as below average. Percentiles between 2 and 8 classify her performance as low. Percentiles below 1 classify her performance as extremely low.

1. Torres VL, Vila-Castelar C, Bocanegra Y, et al. Normative data stratified by age and education for a Spanish neuropsychological test battery: Results from the Colombian Alzheimer's prevention initiative registry. *Applied Neuropsychology: Adult*. 2021;28(2):230-244.

Supplementary Table 2. Description of Tau Pathology
**Frontal lobe:** Very few neurofibrillary tangles without a layering pattern. There was mainly neuropil thread as well as dystrophic neurites, although in a small amount, evenly distributed across the cerebral cortex. There was no perivascular or subpial immunoreactivity.
**Temporal lobe:** Very few neurofibrillary tangles without a layering pattern. There was mainly neuropil thread (NPT) as well as dystrophic neurites (DNs). NPT and DNs compromise layer II and the upper part of layer III as well as the infragranular layer with a very low presence in the space in between. There was no perivascular or subpial immunoreactivity.
**Inferior parietal lobe:** Very few neurofibrillary tangles without a layering pattern. There was mainly neuropil thread (NPT) as well as dystrophic neurites (DNs). NPT and DNs compromise layer II and the upper part of layer III as well as the infragranular layer with a very low presence in the space in between. NPT and DNs were more abundant in the infragranular layers. There was no perivascular or subpial immunoreactivity.
**Occipital lobe:** Very few neurofibrillary tangles (NFT), with an increased presence of NFT in the V layer. There was mainly neuropil thread (NPT) as well as dystrophic neurites (DNs). NPT and DNs compromise layer II and the upper part of layer III as well as the infragranular layer with a very low presence in the space in between. NPT and DNs were more abundant in the infragranular layers. There was no perivascular or subpial immunoreactivity.
**Cerebellum:** No lesions were visible.
**Midbrain/Pons:** Low Tau immunoreactivity with a preponderance of neurofibrillary tangles across the cerebral cortex and greater density of DNs in the subpial region. This low immunoreactivity was not associated to neuronal loss given that neuronal somas were evenly distributed between the bundles of the longitudinal and transverse pontine fibres, with no qualitative decrease in their number.
**Medulla oblongata:** Low Tau immunoreactivity. Pre-tangle formation in the cell bodies of the olivary complex.
**Hippocampus:** Neurofibrillary tangles across Amon´s horn with a less density in the CA3 region, frequent dystrophic neurites in CA1. Tau immunoreactivity in the Subiculum comprises mainly NPT without NFT. Tau immunoreactivity was very low in the Pre-subiculum. NPT and DNs compromise layer II and the upper part of layer III as well as the infragranular layer with a very low presence in the space in between. NPT and DNs were more abundant in the infragranular layers. There was no perivascular or subpial immunoreactivity.
**Amygdala:** Homogenous distribution of NFT and DN over de nucleus. There was no perivascular or subpial immunoreactivity.
**Insula:** Very few neurofibrillary tangles without a layering pattern. There was mainly neuropil thread in the cortex. Dystrophic neurites were seen in the withe matter beneath the cerebral cortex. There was no perivascular or subpial immunoreactivity.
**Cingulate Gyrus:** Neuropil thread across all cerebral cortex, more abundant in the infragranular layer. Neurofibrillary tangles were restricted to the V layer.
**Lenticular nucleus:** Neuropil thread evenly distributed across the nucleus with spares dystrophic neurites. No NFTs were visible.
**Caudate nucleus:** Neuropil thread evenly distributed across the nucleus with spares dystrophic neurites. No NFTs were visible.
**Hypothalamus:** Neuropil thread evenly distributed across the nucleus with spares dystrophic neurites. No NFTs were visible.

Supplementary Table 3. Description of Aβ Pathology
**Frontal lobe:** Profuse accumulation of Aβ in the form of diffuse plaque through the cerebral cortex without a particular layer distribution. Sparse cored plaques were scattered in the supragranular layers. Frequent neuritic plaques and scare cored plaques were present in the VI layer and the adjacent withe matter. There was no evidence of cerebral amyloid angiopathy neither in the meningeal nor in the parenchymal vessels.
**Temporal lobe:** Profuse accumulation of Aβ in the form of diffuse plaque through the cerebral cortex excluding most of the layer II where cored plaques were un-evenly distributed giving a “patchy” appearance to the supragranular segments. Frequent neuritic plaques and scare cored plaques were present in the VI layer and the adjacent withe matter. There was no evidence of cerebral amyloid angiopathy neither in the meningeal nor in the parenchymal vessels.
**Inferior parietal lobe:** Profuse accumulation of Aβ in the form of diffuse plaque through the cerebral cortex excluding most of the layer II where cored plaques and spare neuritic plaques were un-evenly distributed giving a “patchy” appearance to the supragranular segments. Frequent neuritic plaques and scare cored plaques were present in the VI layer and the adjacent withe matter. There was no evidence of cerebral amyloid angiopathy neither in the meningeal nor in the parenchymal vessels.
**Occipital lobe:** Frequent neuritic plaques and scare cored plaques were present in the supragranular layers and adjacent withe matter with diffuse plaques restricted to the subpial and layer I segments. Profuse accumulation of Aβ in the form of diffuse plaque in the infragranular layers with scattered cored plaques. Some circumferential Aβ deposition in the meningeal vessels. There was no evidence of cerebral amyloid angiopathy neither in the parenchymal vessels.
**Cerebellum:** Deposition of Aβ mainly in the form of diffuse plaques and scant cored plaques in the granular layer. Occasional cored plaques were found in the molecular layer. There was no evidence of Aβ deposition in the Purkinje layer. Widespread circumferential Aβ deposition in the meningeal vessels. There was no evidence of cerebral amyloid angiopathy neither in the parenchymal vessels.
**Midbrain/Pons:** *Midbrain:* Diffuse plaques were clustered around the dorsal and lateral boundaries of the tegmental region, as well as the ventral aspect of the peduncular regions. *Pons:* Diffuse plaques were evenly distributed through the tegmental and basilar regions.
**Medulla oblongata:** In the ventral aspect, large diffuse plaques were clustered around the olivary complex. Toward de the dorsal aspect the size of the diffuse plaques decreases, and small cored plaques were seen.
**Hippocampus:** There was Aβ deposition across the different hippocampal subfields with variations in the type of plaques to be found. In *CA3*, there were predominantly cored plaques with scant neuritic plaques. In *CA2* was spare from Aβ burden. Cored plaques as well as large diffuse plaques affect *CA1*. Mainly small diffuse plaques were visible in the *Subiculum.* In the *Pre-subiculum,* there was a profuse accumulation of Aβ in the form of diffuse plaque through the cerebral cortex without a particular layer distribution. In the *Entorhinal Cortex,* there were diffuse plaques in the subpial and layer I segments whereas from layer II through VI there were cored, as well as neurites plaques. There was no evidence of cerebral amyloid angiopathy neither in the meningeal nor in the parenchymal vessels.
**Amygdala:** Cored and neurites plaques were evenly distributed across the nucleus. Scant deposition of Aβ in the walls of the arterioles.
**Insula:** Profuse accumulation of Aβ in the form of diffuse plaque through the cerebral cortex excluding most of the layer II where cored plaques were un-evenly distributed giving a

"patchy" appearance to the supragranular segments. Frequent neuritic plaques and scare cored plaques were present in the VI layer and the adjacent withe matter. There was no evidence of cerebral amyloid angiopathy neither in the meningeal nor in the parenchymal vessels.
**Cingulate Gyrus:** Profuse accumulation of Aβ in the form of diffuse plaque through the cerebral cortex without a particular layer distribution. Sparse cored plaques were scattered in the supragranular layers. Frequent neuritic plaques and scare cored plaques were present in the VI layer and the adjacent withe matter. There was no evidence of cerebral amyloid angiopathy neither in the meningeal nor in the parenchymal vessels.
**Lenticular nucleus:** Profuse accumulation of Aβ in the form of diffuse plaque through the Putamen, whereas both segments of the Globus Pallidus were spare from plaques except for a cluster of diffuse plaque restricted to the medial medullary lamina.
**Caudate nucleus:** Profuse accumulation of Aβ in the form of diffuse and cored plaques through the putamen and caudate nucleus.
**Hypothalamus:** Diffuse plaques evenly distributed across the nucleus with spare cored plaques

Supplementary Table 4. Cell sequencing metrics			
**Area**	**Reads / Cell**	**Genes / Cell**	**UMI / Cell**
Frontal cortex	39958	2450	5054
Hippocampus	41435	2314	5071
Occipital lobe	33067	1246	2463

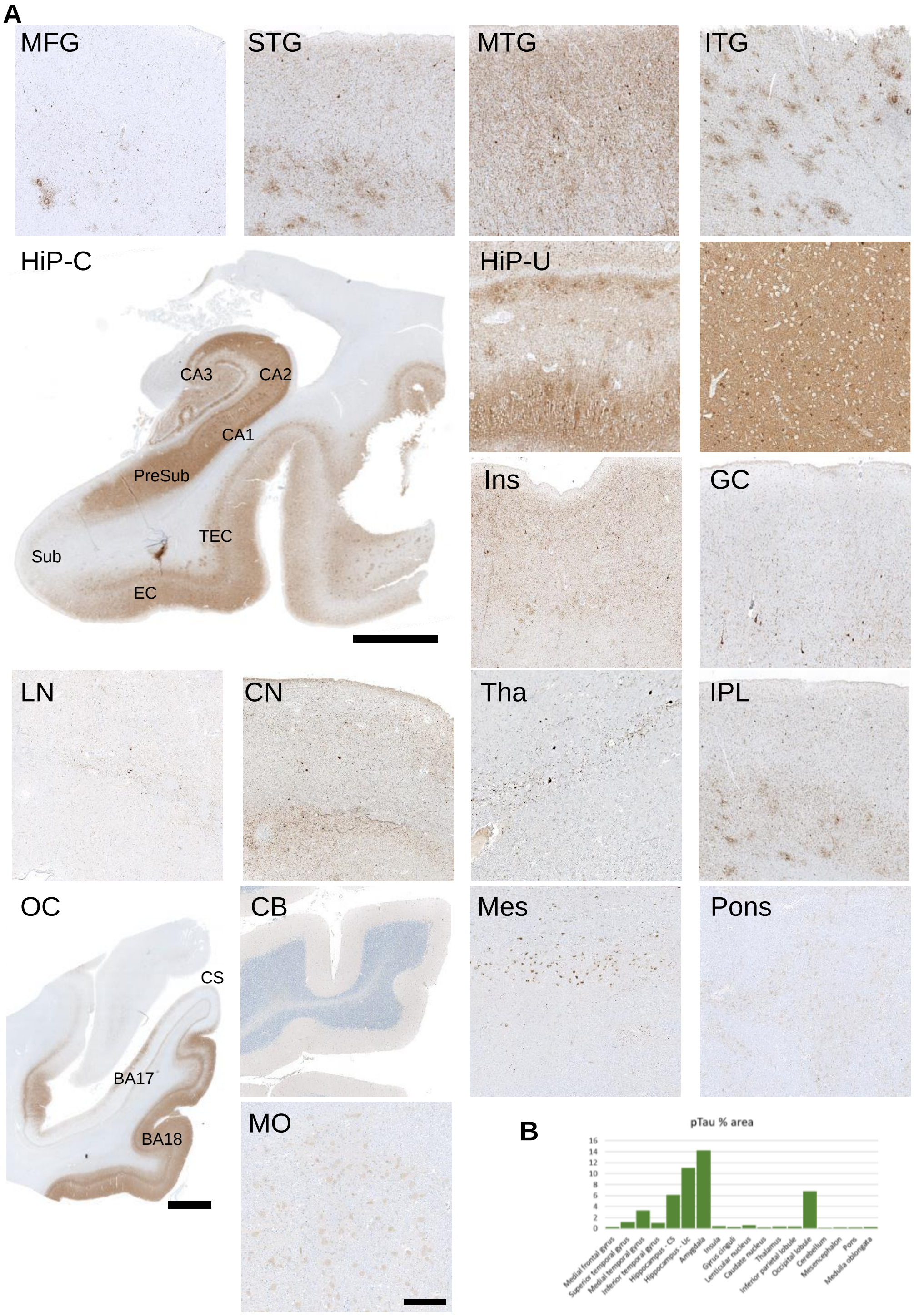

Supp. Fig. 1: pTau pathology characterization of an ADAD PSEN1 E280A APOE3ch homozygote case. A. Representative images of IHC staining with using the AT8 antibody for pTau. MFG = Medial frontal gyrus, STG = Superior temporal gyrus, MTG = Medial temporal gyrus, ITG = Inferior temporal gyrus, HiP-C = Hippocampus – CS, Hip-U = Hippocampus – Uncus, Amy = Amygdala, Ins = Insula, GC = Gyrus cinguli, LN = Lenticular nucleus, CN = Caudate nucleus, Tha =

Thalamus, IPL = Inferior parietal lobule, OC = Occipital lobule, CB = Cerebellum, Mes = Mesencephalon, Pons = Pons, MO = Medulla oblongata. Bar=500 μm. B. Bar graph for the percentage of pTau signal affecting the studied areas.

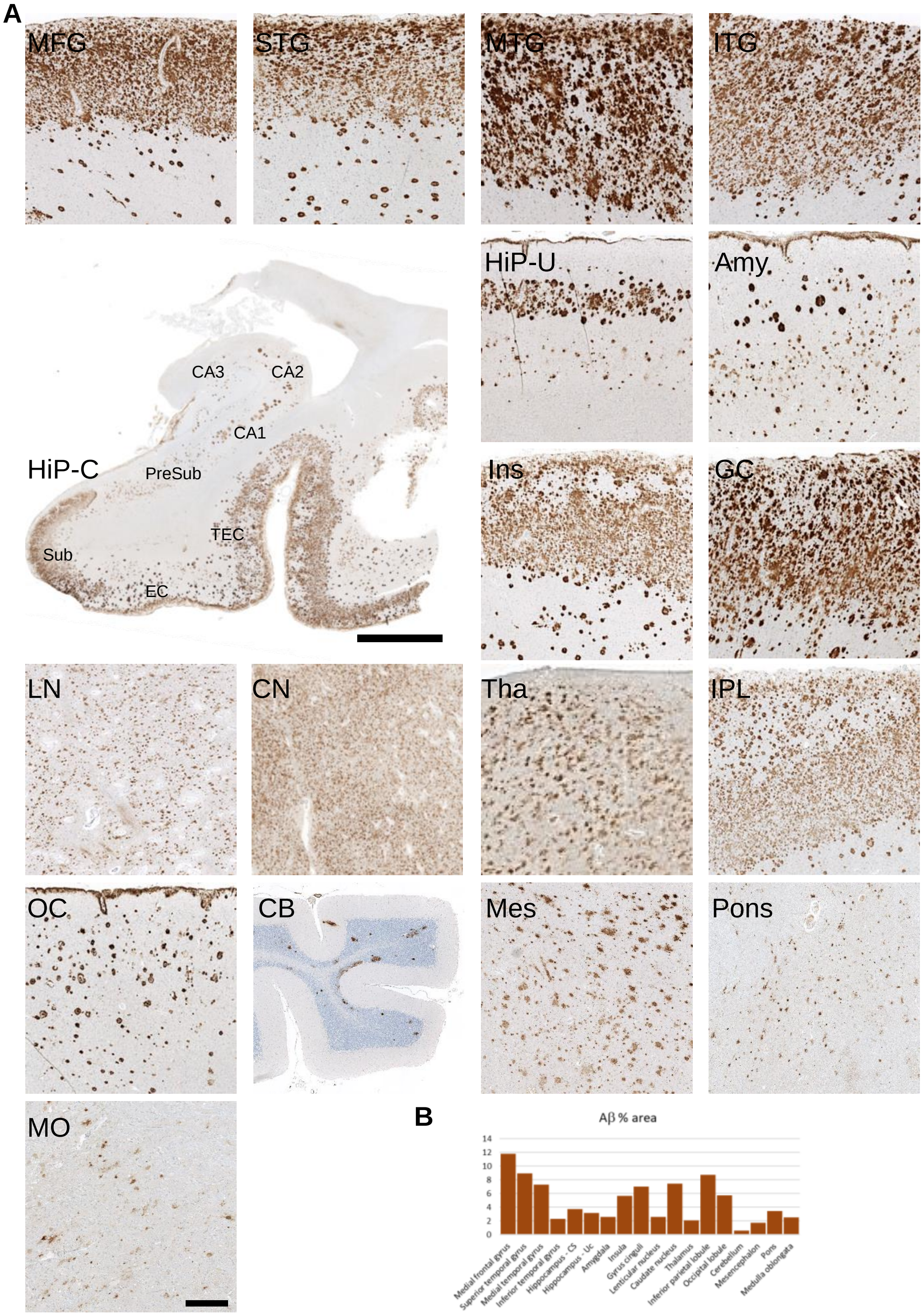

Supp. Fig. 2: Aβ pathology characterization of an ADAD PSEN1E280A APOE3ch homozygote case. A. Representative images of IHC staining with using the BAM-10 antibody for Aβ. MFG = Medial frontal gyrus, STG = Superior temporal gyrus, MTG = Medial temporal gyrus, ITG = Inferior temporal gyrus, HiP-C = Hippocampus – CS, CA3 = Cornu Ammonis 3, CA2 = Cornu Ammonis 2, CA1 = Cornu Ammonis 1, PreSub = PreSubiculum, Sub = Subiculum, EC = Entorhinal Cortex, TEC = Transentorhinal Cortex,

Hip-U = Hippocampus – Uncus, Amy = Amygdala, Ins = Insula, GC = Gyrus cinguli, LN = Lenticular nucleus, CN = Caudate nucleus, Tha = Thalamus, IPL = Inferior parietal lobule, OC = Occipital lobule, CB = Cerebellum, Mes = Mesencephalon, Pons = Pons, MO = Medulla oblongata. Bar=500 μm. B. Bar graph for the percentage of Aβ signal affecting the studied areas. Specifically, Aβ pathology distribution showed stronger signal in all studied cortices, with frontal cortex, temporal cortex, and gyrus cinguli presenting with uniformly diffuse plaques together with core plaques in subcortical white matter. Inferior parietal lobe and insula showed diffuse subpial plaques, with larger diffuse plaques in infragranular cortical layers and core plaques in subcortical white matter. Subcortical structures presented small diffuse plaques with some intraneuronal signal in mesencephalon, pons and medulla oblongata and Cerebellum presented with diffuse plaques in the granular layer. Finally, Aβ aggregates were heterogeneous in hippocampus and parahippocampal structures, with large mature and core plaques in CA1, CA2 and entorhinal cortex, and diffuse plaques in subiculum and transentorhinal cortex.

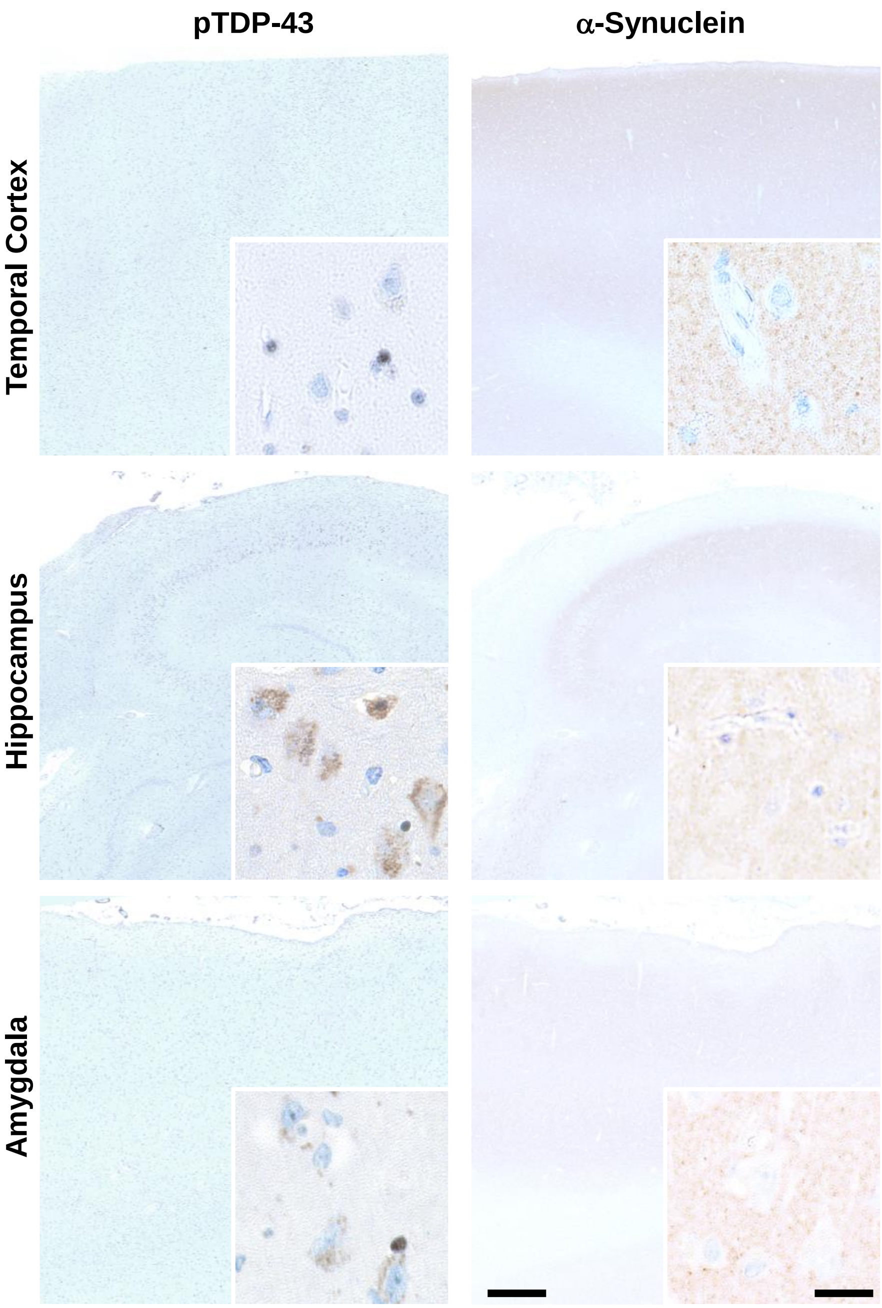

Supp. Fig. 3: pTDP-43 and α-Synuclein staining ADAD PSEN1E280A APOE3ch homozygote case. Representative images of the temporal cortex, hippocampus and amygdala. Insets depict neurons in each area, with diffuse granular deposits for pTDP-43 and no deposits of α-Synuclein. Bars = 500 μm, in insets = 25 μm.

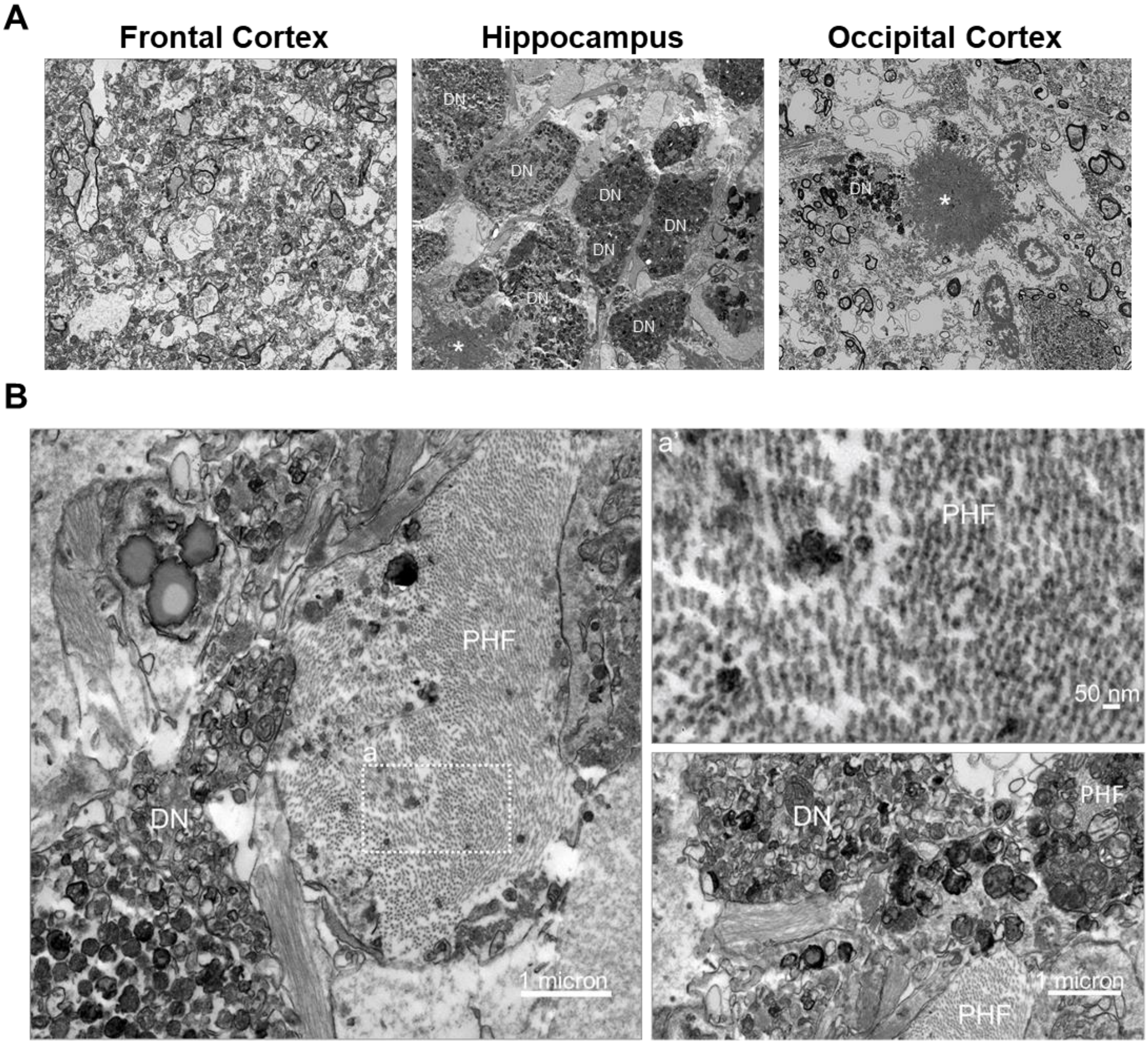

Supp. Fig 4: EM analysis of pTau pathology of the hippocampus of an ADAD *PSEN1*E280A APOE3ch homozygote case. A. Representative electron micrographs of different brain areas. Ultrastructural analysis revealed neuritic plaques in hippocampus and occipital cortex, characterized by the presence of Aβ deposits (*) and large dystrophic neurites (DN) filled with degenerating organelles. Paired helical filaments were also observed. B. Electron micrographs of a neuritic plaque in the hippocampus showing extensive neuritic dystrophy (DN) and 20 nm wide paired helical filaments (PHF). The high magnification image (a') corresponds to the same area indicated by the white square (a).

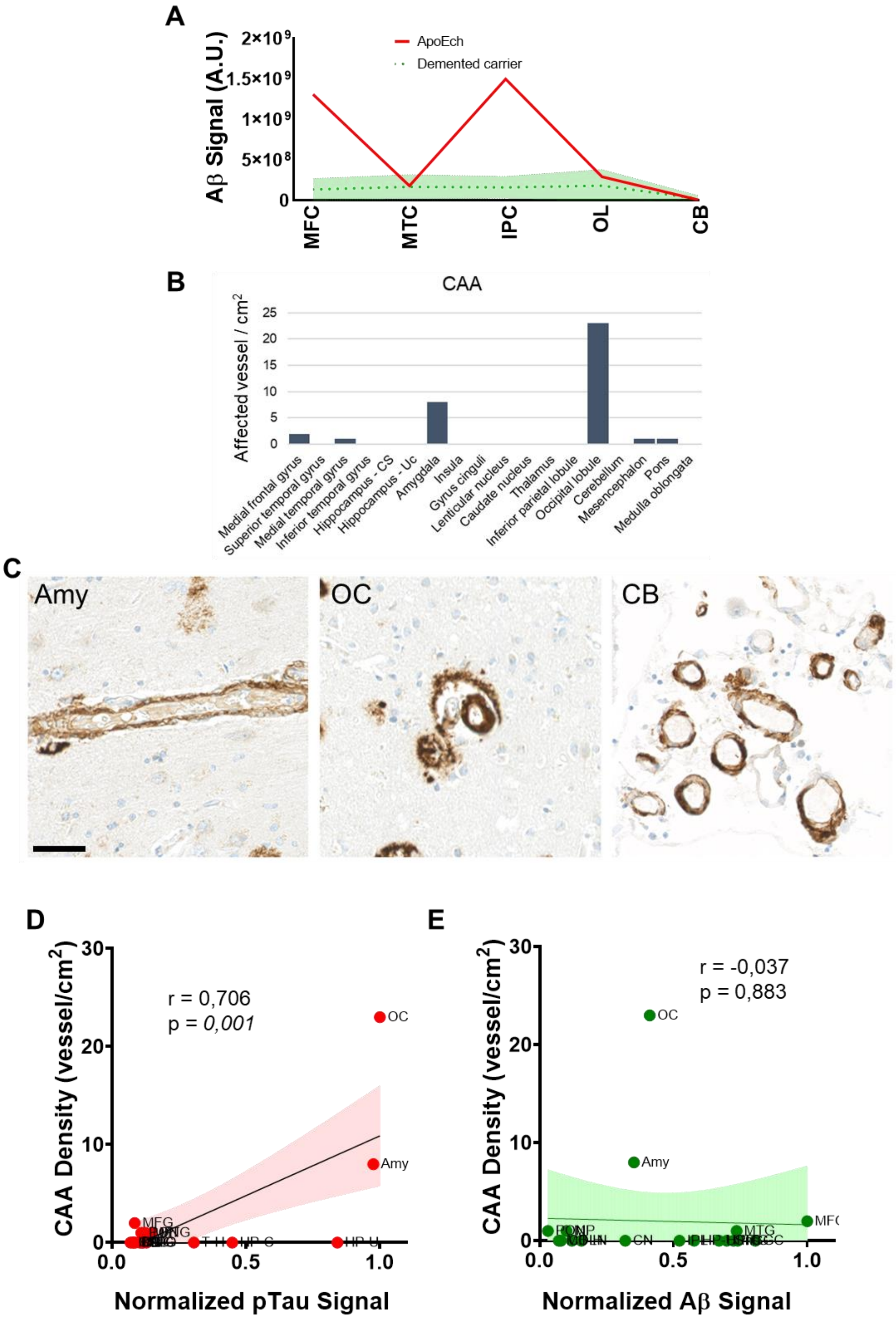

Supp. Fig 5: Aβ pathology and CAA characterization of an ADAD *PSEN1*E280A APOE3ch homozygote case. A. Distribution area plot showing Aβ integrated density signal in cortical areas in APOEch homozygote (red line) relative to unimpaired (blue) and

impaired (green) PSEN1-E280A carriers. B. Bar graph for CAA density in studied brain areas. C. Representative images of CAA affected vessels in the two areas with higher CAA parenchymal density, Amygdala (Amy) and Occipital Cortex (OC), together with the only area presenting with leptomeningeal CAA, Cerebellum (CB). Bar = 50 μm. D. Correlation scatterplot depicting a statistically significant positive correlation between pTau signal intensity and CAA density. E. Correlation scatterplot depicting Aβ signal intensity and CAA density.

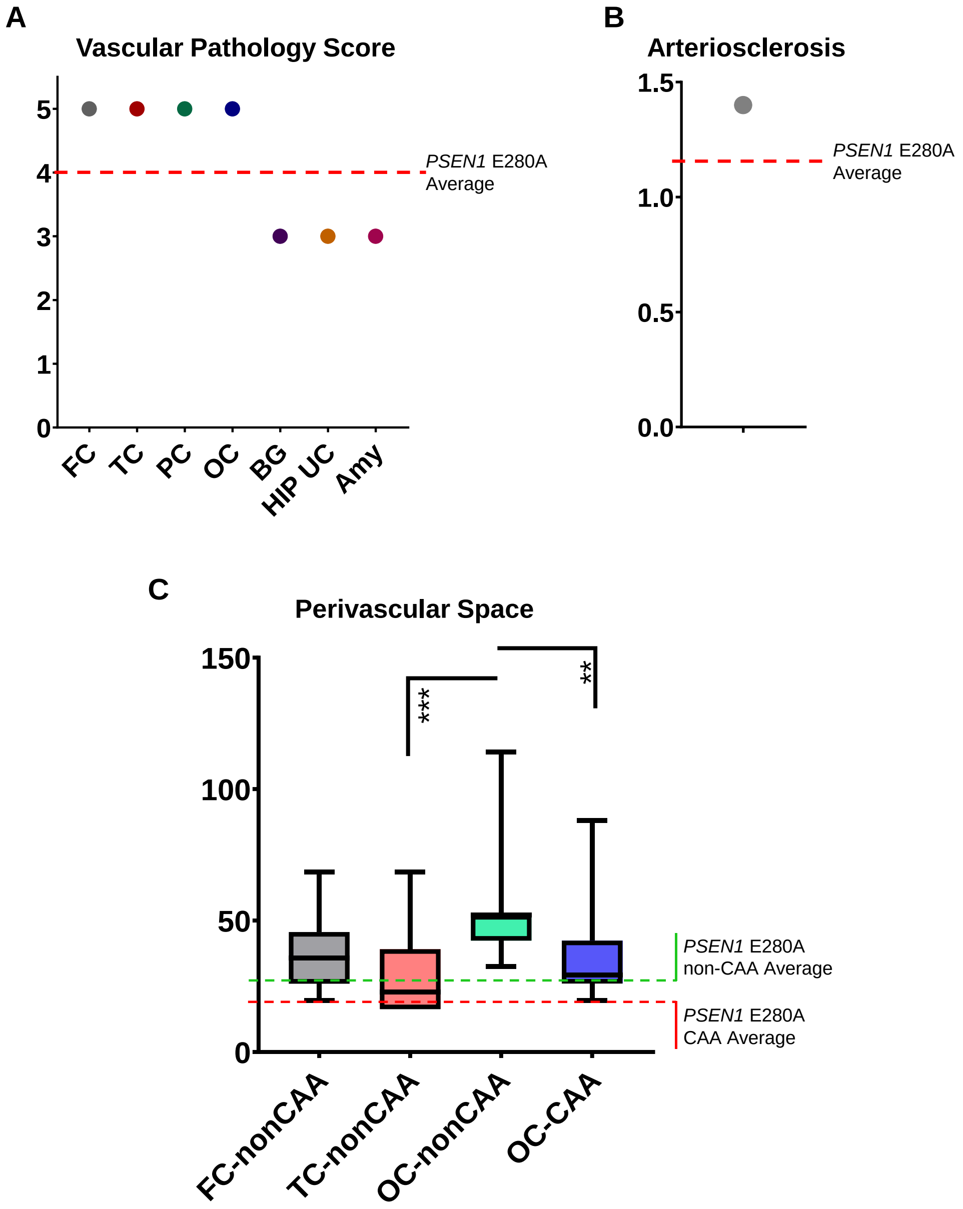

Supp. Fig. 6: Vascular pathology assessment on an ADAD PSEN1E280A APOE3ch homozygote case. A. Dot plot for vascular pathology scores for cortical and limbic regions, cortices showed higher scores due to the presence of arteriosclerosis, increased perivascular spaces and microinfarcts. B. Average score of arteriosclerosis in this case. C. Box and whiskers plot for perivascular spaces in microvessels with and without CAA pathology. Dashed lines show average values for these features as reported in Littau JL et al. 2022 (PMID: 35695802).

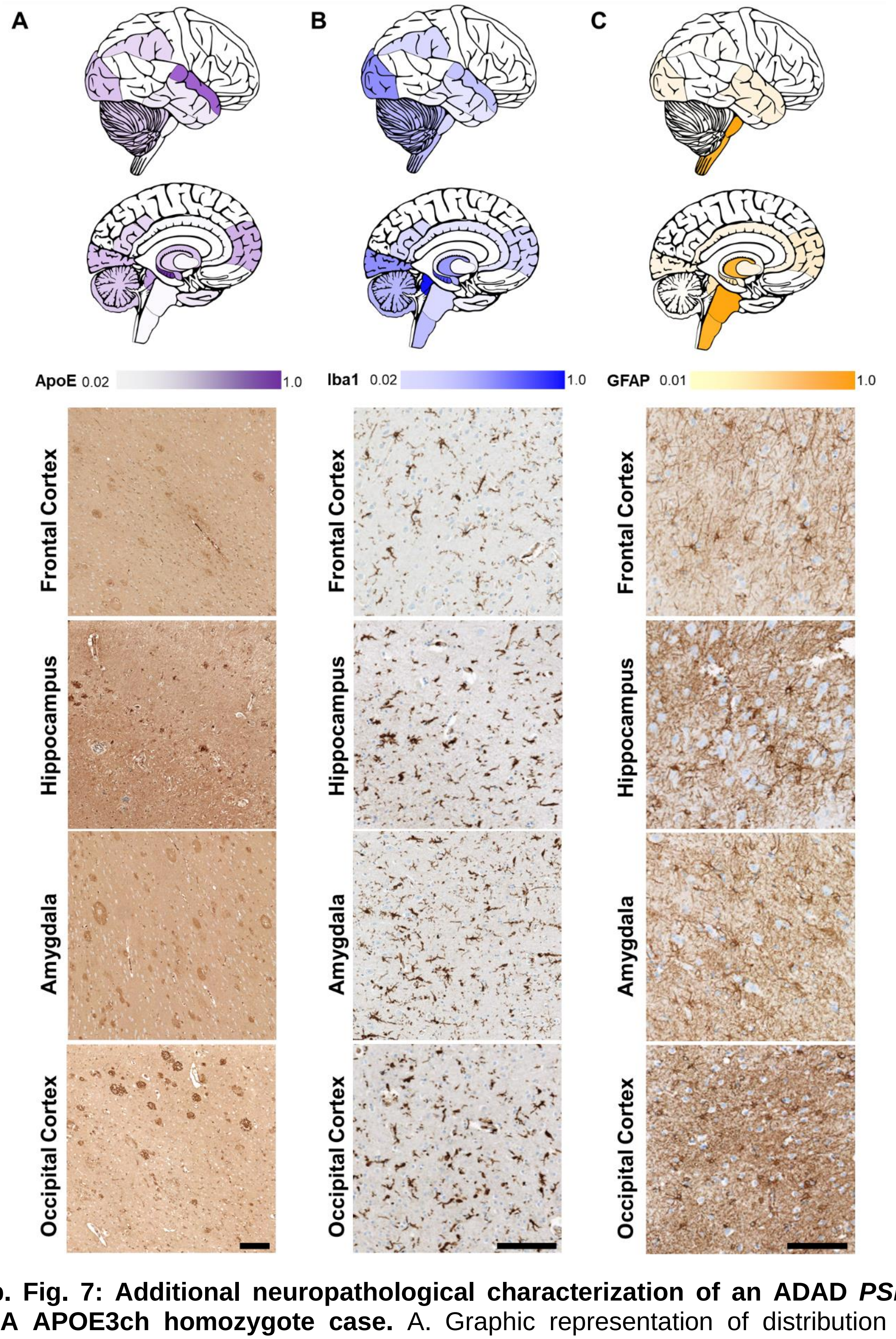

Supp. Fig. 7: Additional neuropathological characterization of an ADAD *PSEN1* E280A APOE3ch homozygote case. A. Graphic representation of distribution and intensity of ApoE signal with normalized lower and maximum values represented in blue

and yellow intensities, respectively. Representative images of ApoE staining can be seen below for cortical and limbic structures. ApoE staining shows stronger signal in the shape of plaques, with stronger signal in hippocampus and occipital cortex. B. Graphic representation of distribution and intensity of Iba1 signal with normalized lower and maximum values represented in blue and yellow intensities, respectively. Representative images of Iba1 staining can be seen below for cortical and limbic structures. Iba1 stained microglia look more abundant, larger, and more ramified in hippocampus and occipital cortex. C. Graphic representation of distribution and intensity of GFAP signal with normalized lower and maximum values represented in blue and yellow intensities, respectively. Representative images of GFAP staining can be seen below for cortical and limbic structures. GFAP stained astrocytes look more abundant, larger, and more ramified in hippocampus and occipital cortex. All brain color maps represent the general localization of the staining, they do not represent exact anatomical locations.

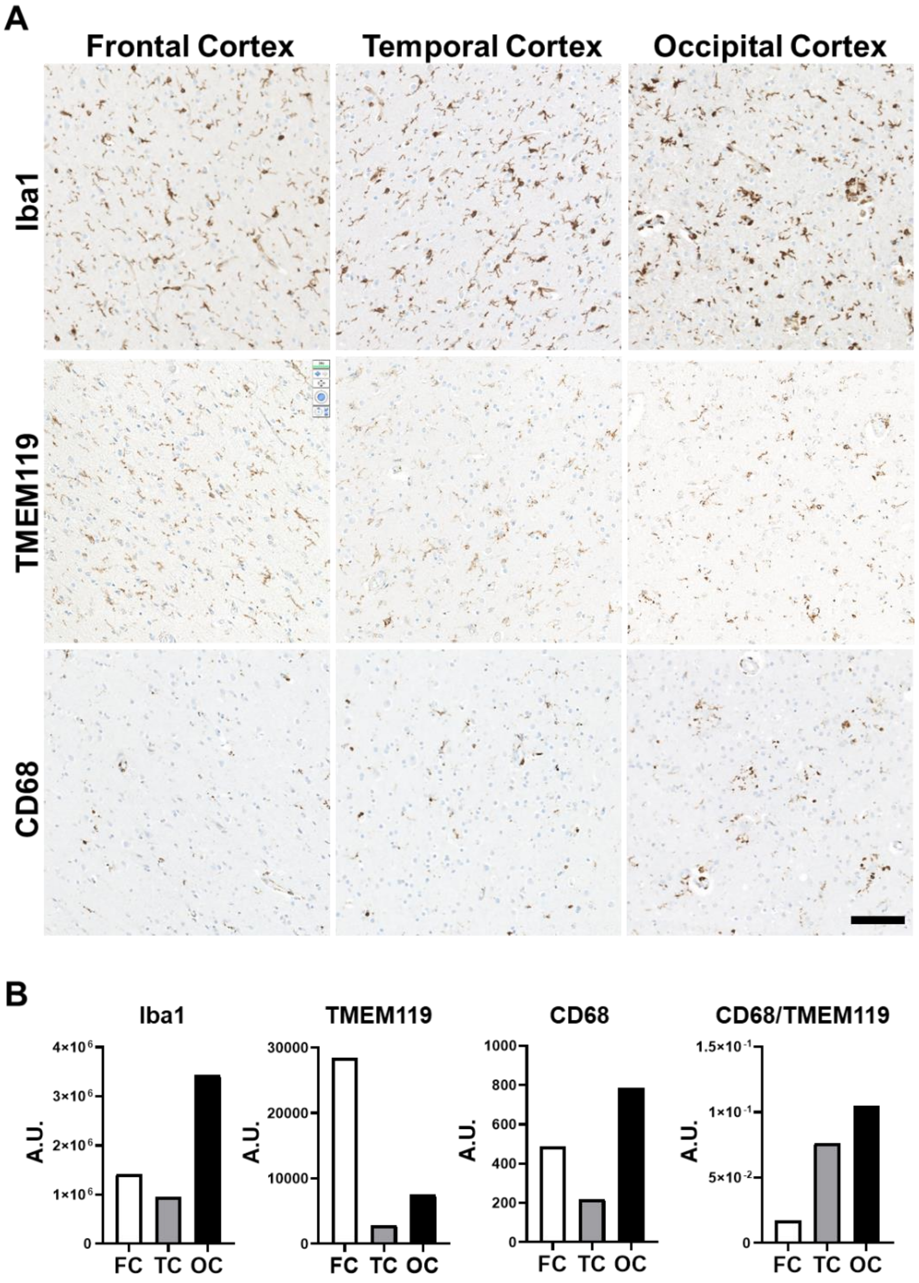

Supp. Fig. 8: Microglial profile of an ADAD *PSEN1* E280A APOE3ch homozygote case. A. Representative pictures of cortical sections stained with IBA1, TMEM119 and CD68. Bar = 100 μm. B. Bar graphs depicting Iba1, TMEM119 and CD68 signal intensity in frontal cortex (FC), temporal cortex (TC) and occipital cortex (OC), together with CD68/TMEM119 ratio as indirect measurement of microglia activation.

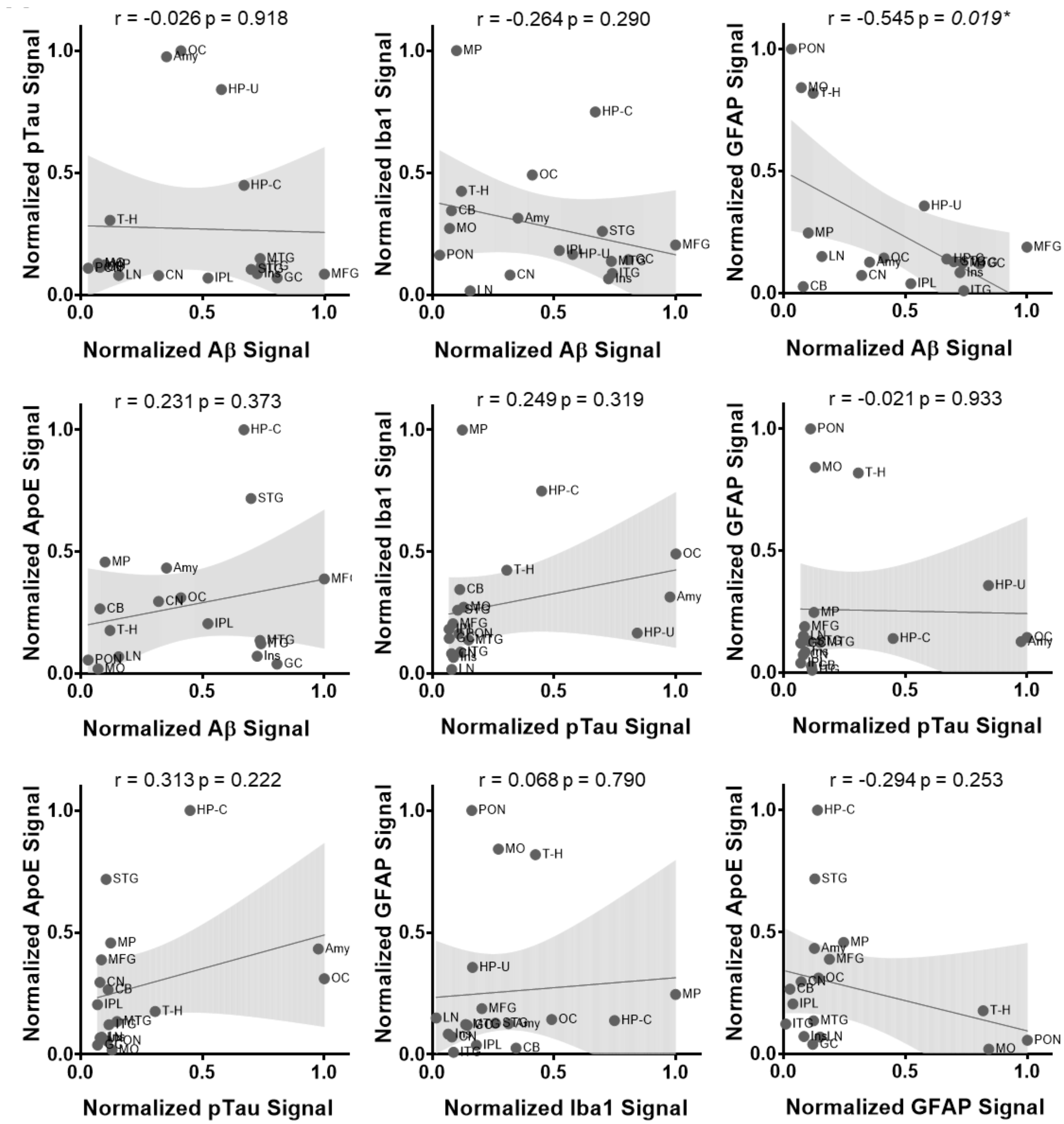

Supp. Fig 9: Correlational analysis between neuropathological variables studied in an ADAD *PSEN1*E280A APOE3ch homozygote case. A. Correlation scatterplots between Aβ, pTau, ApoE, Iba1 and GFAP for all evaluated areas. There was a statistically significant positive correlation between Aβ and GFAP signal. (Supp. Fig. 2, Supp. Fig. 3 and Supp. Fig. 5). $p \leq 0.05$.

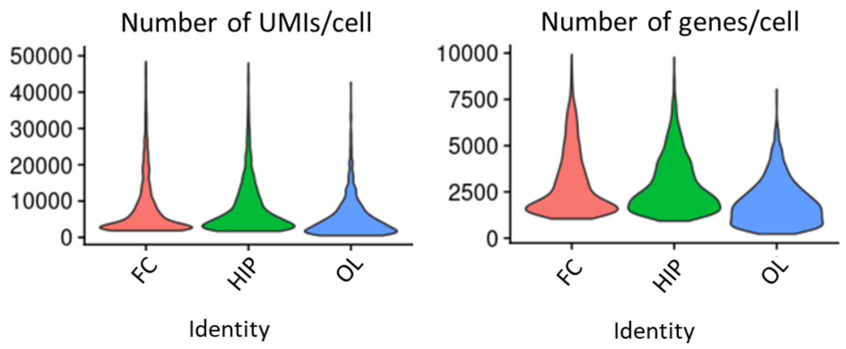

Supp. Fig. 10: sn-RNAseq particles obtained for analysis from an ADAD *PSEN1*E280A APOE3ch. Number of cells and number of genes identified in frontal cortex (FC), hippocampus (HIP) and occipital lobe (OL).

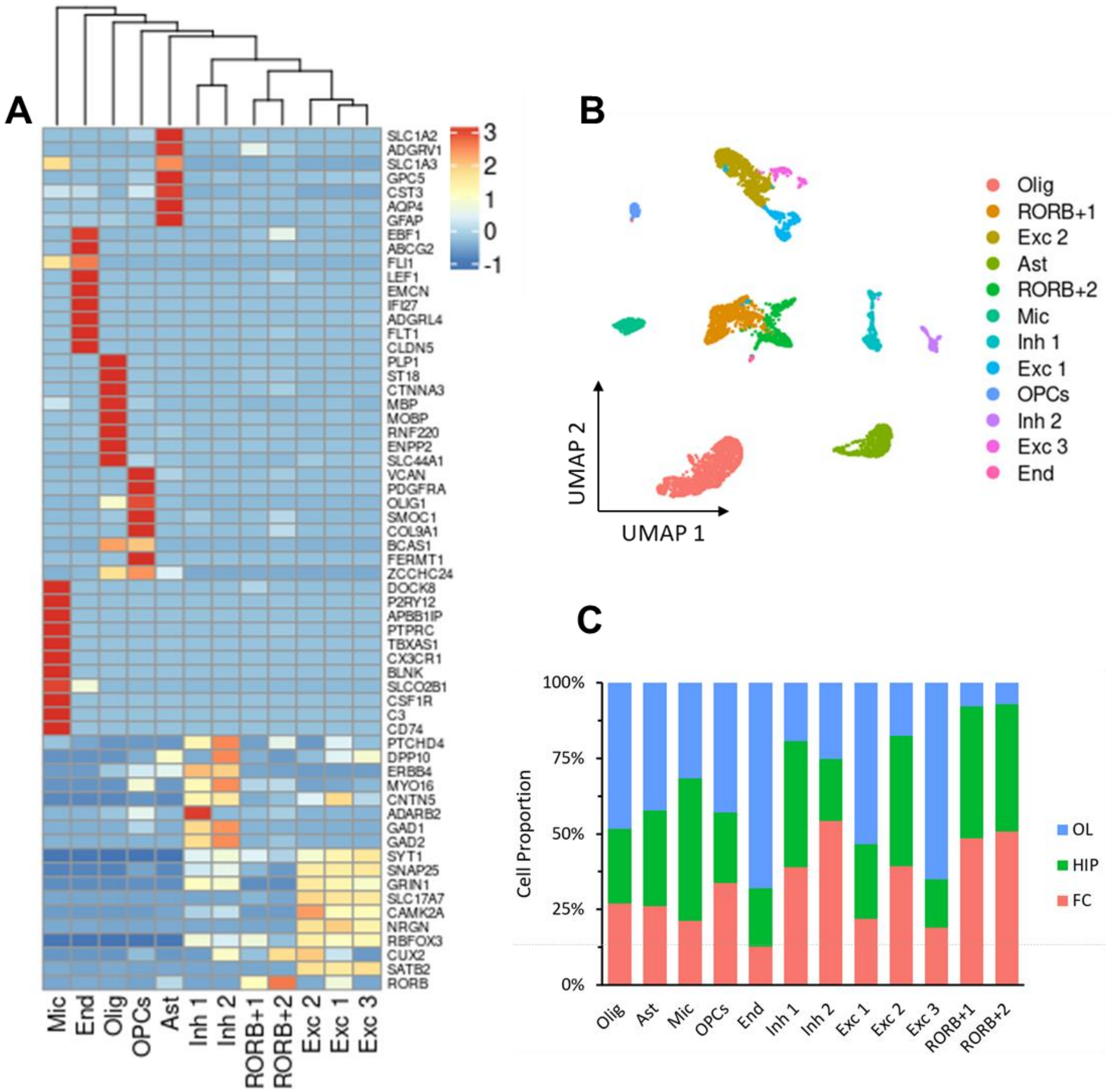

Supp. Fig. 11: Cell type characterization and identification from brain tissue from a *PSEN1*E280A APOE3ch homozygote case. A. Heatmap showing the differential scores (z-scores) for the gene markers used to annotate cell type. B. UMAP visualization showing clustering of single nuclei, colored by cell types. Identified clusters include excitatory neurons (Exc 1, 2 and 3), oligodendrocyte precursor cells (OPCs), microglia (Mic), RORB positive neurons (RORB+ 1 and 2), inhibitory neurons (Inh 1 and 2), endothelial cells (End), oligodendrocytes (Olig) and astrocytes (Ast). C. Proportion of cells from each region in each cell category subcluster. OL: occipital lobe, HIP: hippocampus, FC: frontal cortex.

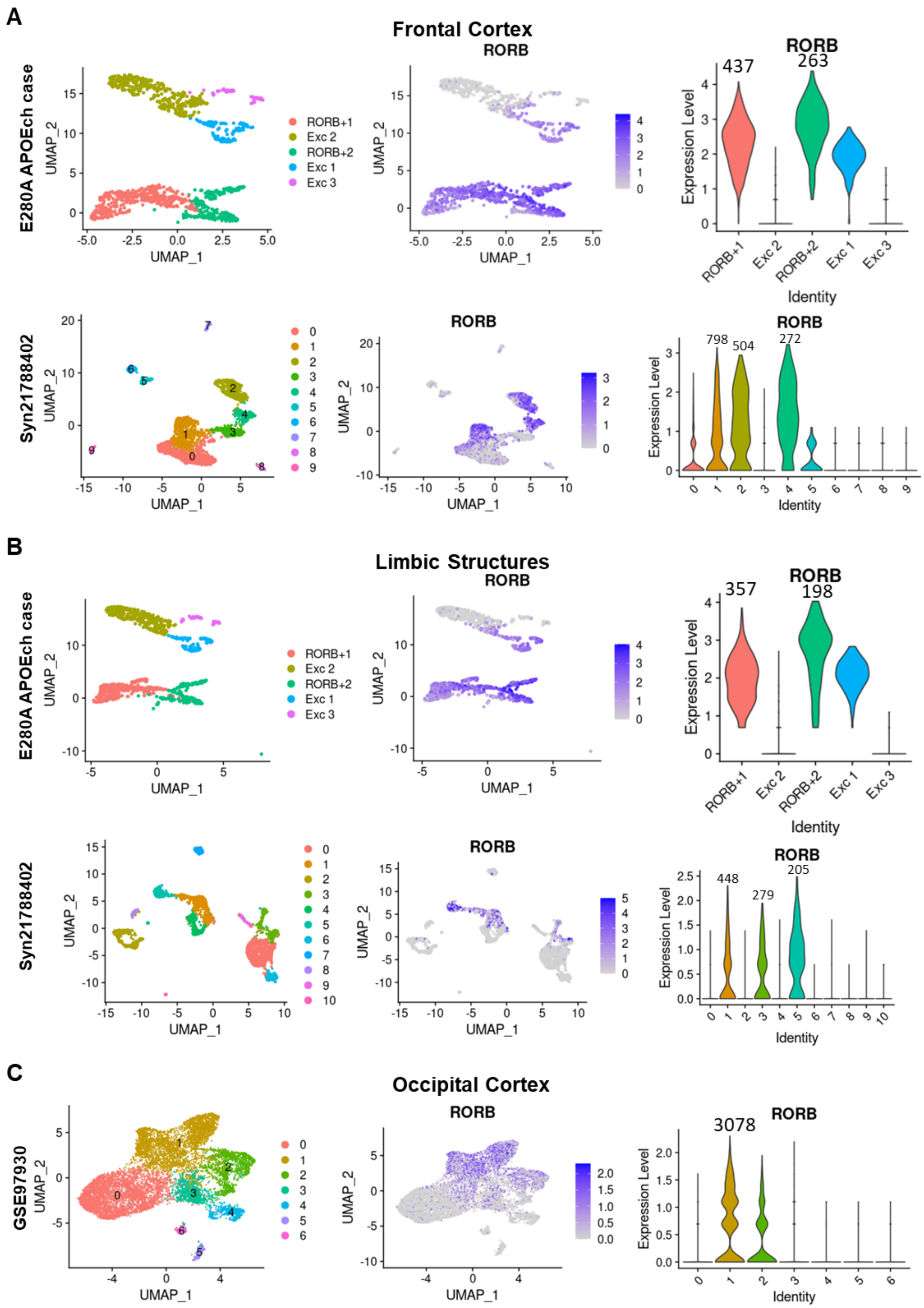

Supp. Fig 12: RORB positive excitatory neurons profile in controls and in an ADAD PSEN1E280A APOE3ch. A. UMAP clusters and violin plots for RORB positive cells as observed in frontal cortex of the Syn21788402 dataset (Leng K et al. 2021) contrasted with RORB positive cells in the APOE3ch case from the same region. B. UMAP clusters

and violin plots for RORB positive cells as observed in the entorhinal cortex of the Syn21788402 dataset (Leng K et al. 2021) contrasted with RORB positive cells in the hippocampus of the APOE3ch case. C. UMAP clusters and violin plots for RORB positive cells as observed in the visual cortex of the GSE97930 dataset (Lale BB et al. 2018), a comparison with the APOE3ch case was not possible because there were not excitatory RORB positive neurons in that region.

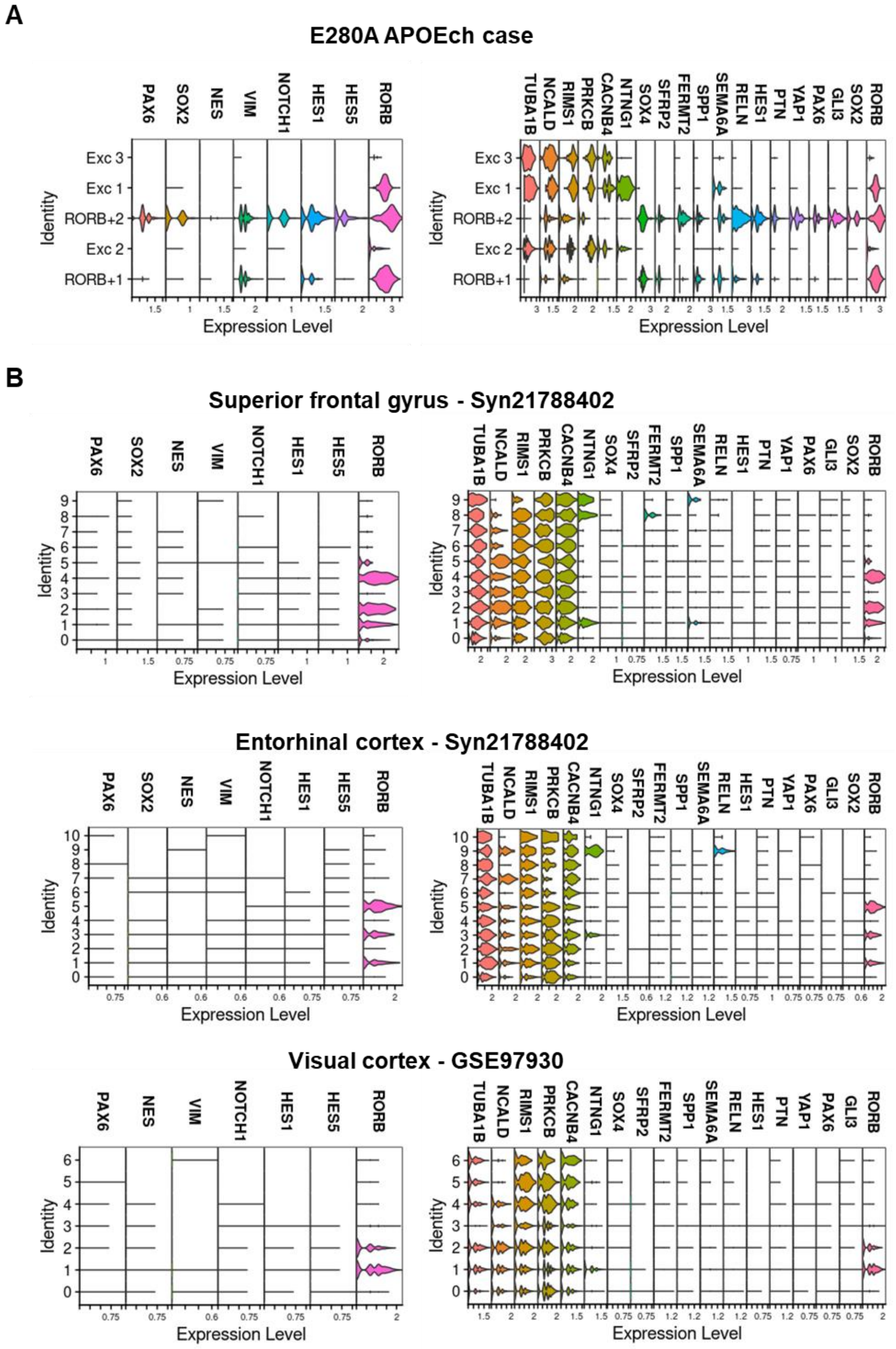

Supp. Fig. 13: Neurodevelopment-associated gene expression in RORB positive neurons clusters in controls and in an ADAD PSEN1E280A APOE3ch case. A. Violin plots depicting neurodevelopment-associated genes identified in the ADAD PSEN1E280A APOE3ch case. B. Violin plots depicting neurodevelopment-associated

genes identified in the control datasets used in the Supp. Fig. 12. Gene onthology analysis showed GO:0048812 and GO:0061564 among the top overrepresented gene sets in the Frontal cortex, GO:0000902 and R-HSA-9675108 among the top overrepresented gene sets in entorhinal cortex from the Syn21788402 dataset. Visual cortex from the GSE97930 dataset showed overrepresentation of GO:0031175, GO:0010975 and GO:0021953 gene sets. The ADAD PSEN1E280A APOE3ch case showed a distinct gene expression profile in contrast to controls.

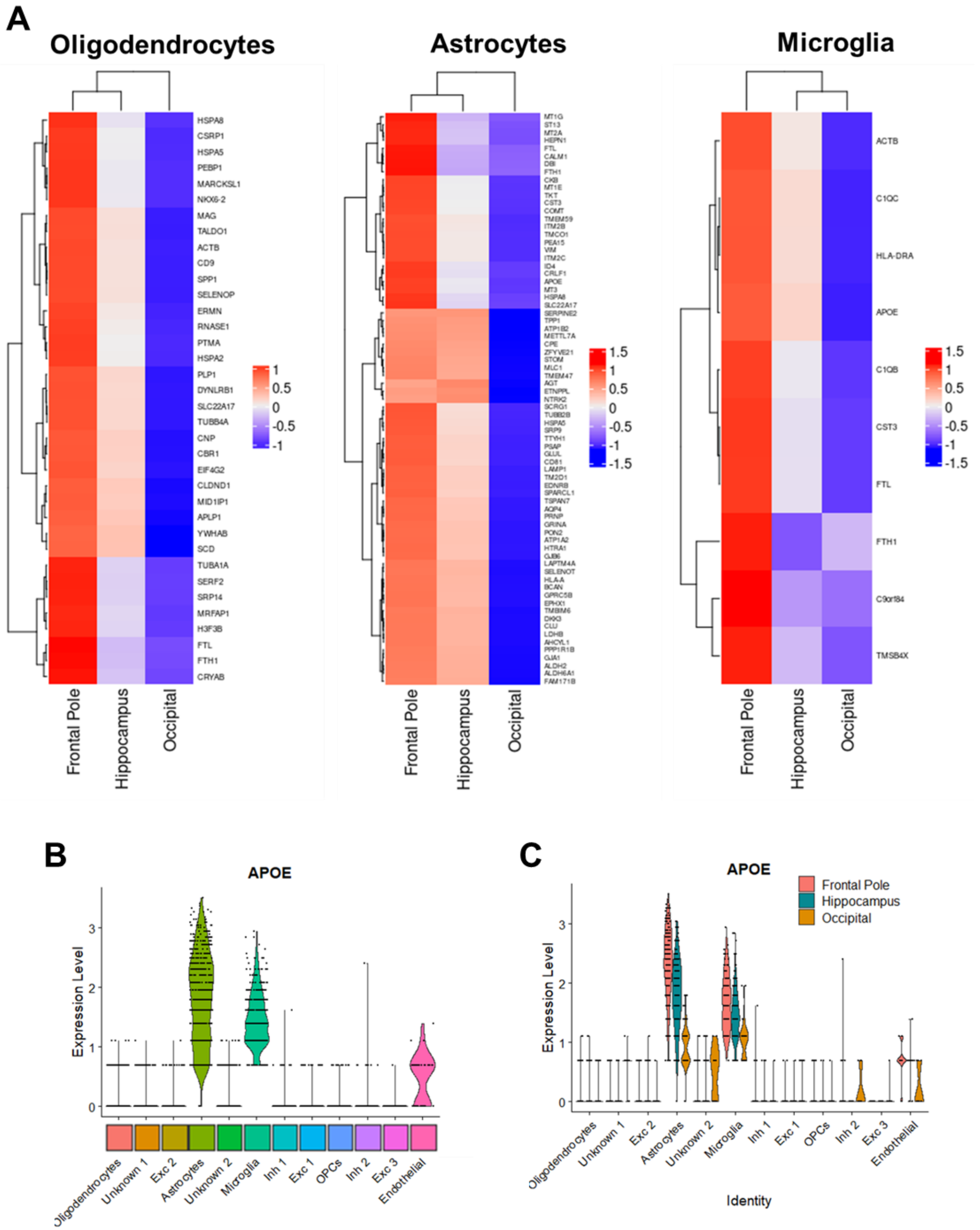

Supp. Fig. 14: Differential gene expression (DGE) in non-neuronal cell subtypes and ApoE selective expression studied in an ADAD *PSEN1*E280A APOE3ch homozygote case. A. Heatmaps depicting top genes with differential expression profiles in oligodendrocytes, astrocytes and microglia. To be noted, APOE was included in the genes detected in astrocytes and microglia. B. Violin plots for APOE expression in the

different cell subtypes detected. C. Violin plots for APOE expression according to brain area in astrocytes and microglia. To be noted, there is a minimal expression threshold for APOE in microglia cells.

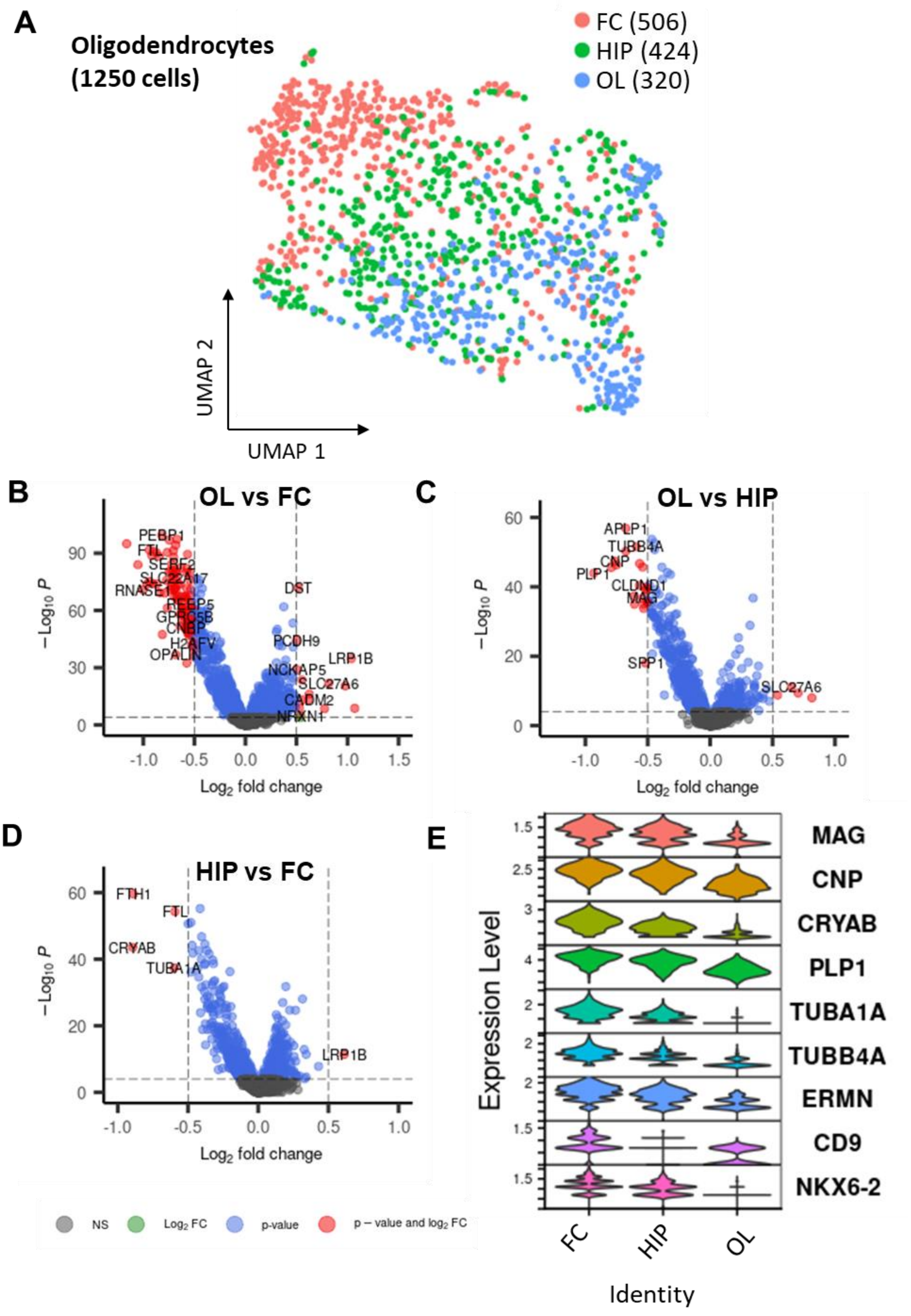
A
Oligodendrocytes (1250 cells)
FC (506)
HIP (424)
OL (320)
UMAP 1
UMAP 2
B
OL vs FC
C
OL vs HIP
D
HIP vs FC
−Log10 P
Log2 fold change
PEBP1
FTL
SERF2
SLC22A17
RNASE1
REEP5
GPRC5B
CNBP
H2AFV
OPALIN
DST
PCDH9
LRP1B
NCKAP5
SLC27A6
CADM2
NRXN1
APLP1
TUBB4A
CNP
PLP1
CLDND1
MAG
SPP1
FTH1
CRYAB
TUBA1A
NS
Log2 FC
p-value
p – value and log2 FC
E
MAG
CNP
CRYAB
PLP1
TUBA1A
TUBB4A
ERMN
CD9
NKX6-2
Expression Level
FC
HIP
OL
Identity

Supp. Fig. 15: DGE in oligodendrocytes from an ADAD *PSEN1*E280A APOE3ch homozygote case. A. Dimesionality reduction scatterplot for oligodendrocytes differentiating between frontal cortex, hippocampus, and occipital cortex. B. Volcano plot for DGE analysis between oligodendrocytes from the occipital versus frontal cortex. C. Volcano plot for DGE analysis between oligodendrocytes from the occipital cortex versus hippocampus. D. Volcano plot for DGE analysis between oligodendrocytes from the hippocampus versus frontal cortex. E. Violin Plots for genes associated with myelination and glial cell differentiation.

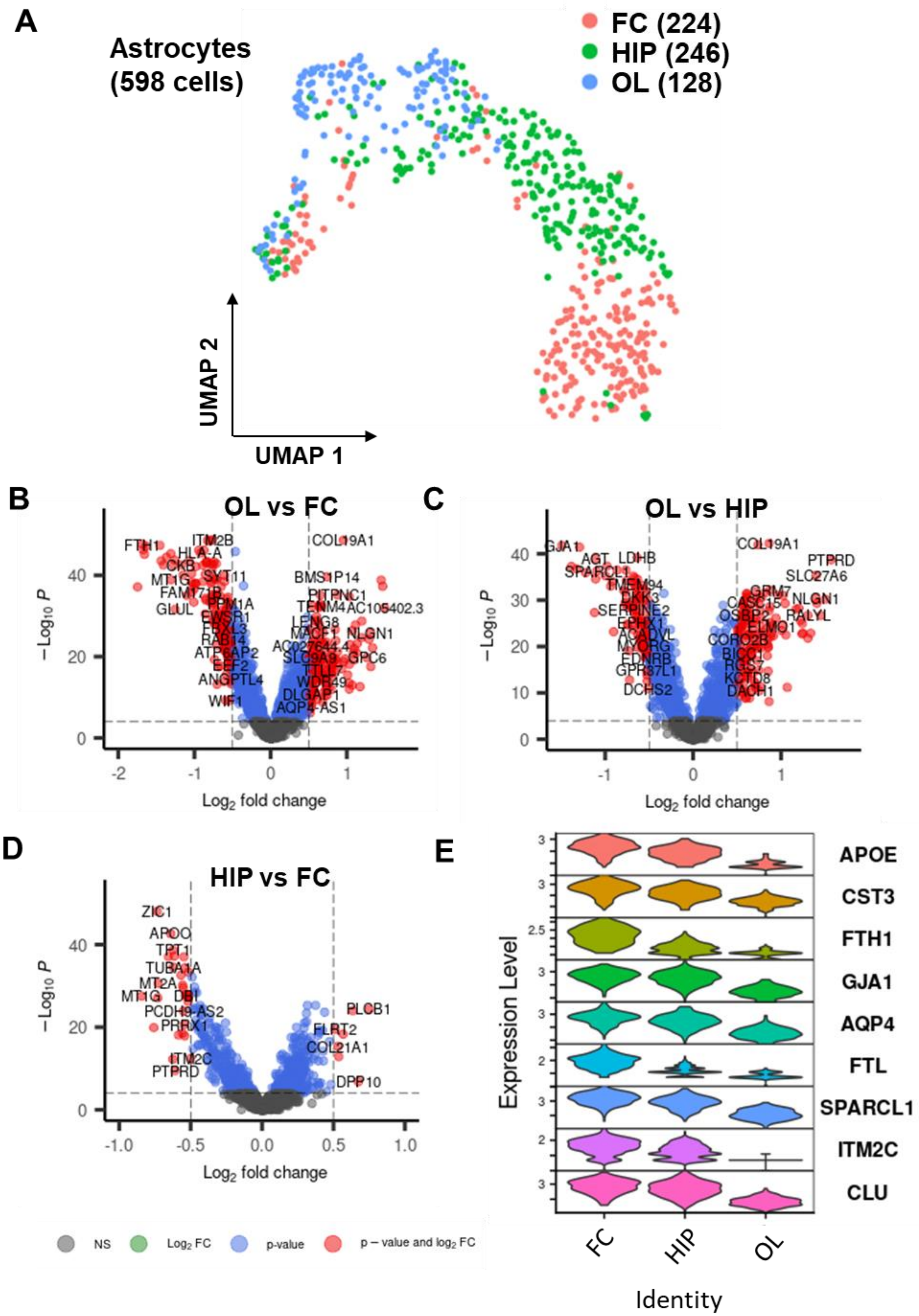
A
Astrocytes
(598 cells)
FC (224)
HIP (246)
OL (128)
UMAP 2
UMAP 1
B
OL vs FC
C
OL vs HIP
D
HIP vs FC
E
APOE
CST3
FTH1
GJA1
AQP4
FTL
SPARCL1
ITM2C
CLU
Expression Level
Identity
FC
HIP
OL
Log2 fold change
NS
Log2 FC
p-value
p – value and log2 FC

Supp. Fig. 16: DGE in astrocytes from an ADAD *PSEN1*E280A APOE3ch homozygote case. A. Dimesionality reduction scatterplot for astrocytes differentiating between frontal cortex, hippocampus, and occipital cortex. B. Volcano plot for DGE analysis between astrocytes from the occipital versus frontal cortex. C. Volcano plot for DGE analysis between astrocytes from the occipital versus hippocampus. D. Volcano plot for DGE analysis between astrocytes from the hippocampus versus frontal cortex. E. Violin plots for top genes identified.

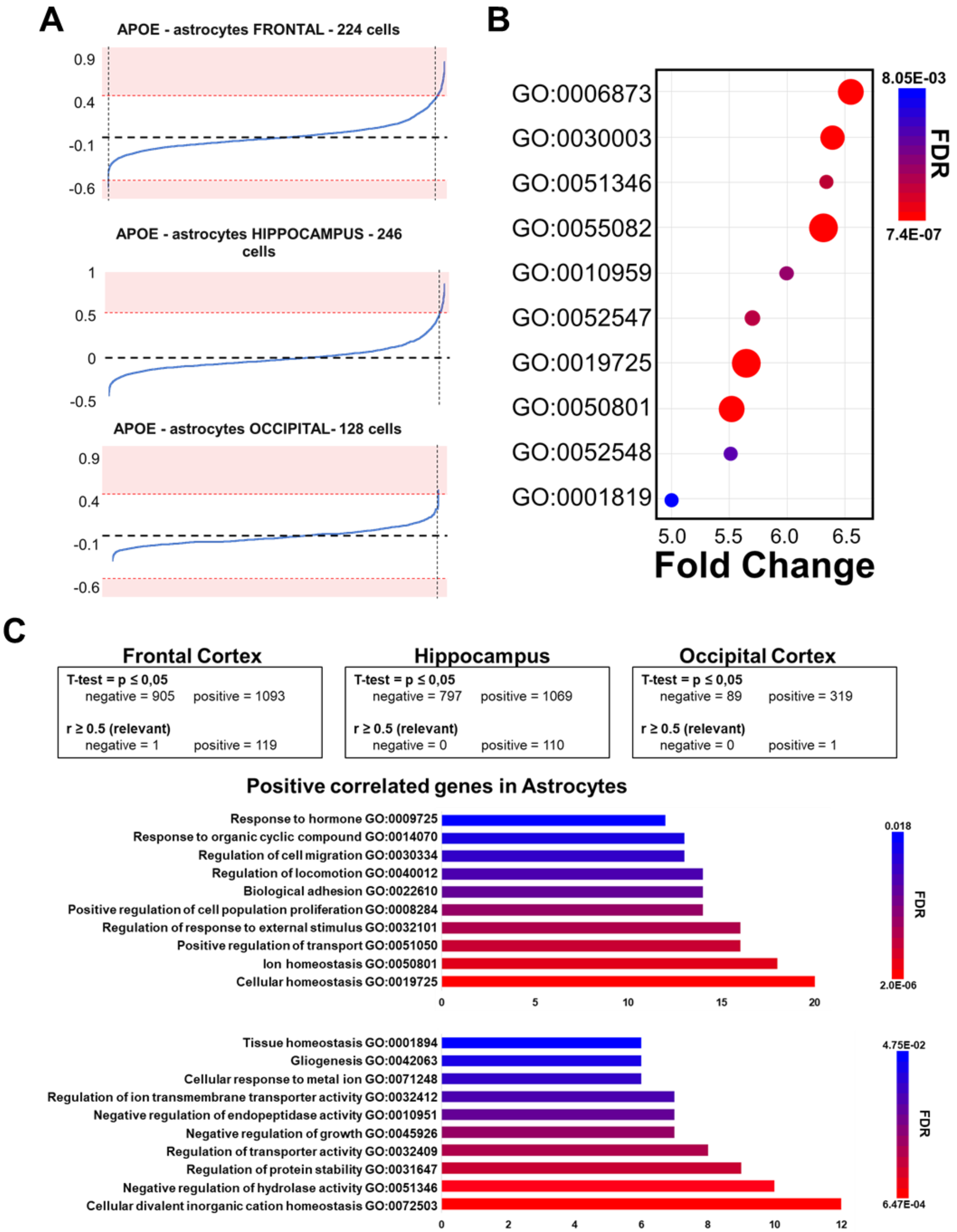

Supp. Fig. 17: Analysis of gene expression correlating with APOE expression in astrocytes of ADAD *PSEN1*E280A APOE3ch homozygote case. A. Correlation distribution of genes in frontal cortex, hippocampus and occipital cortex. Red dashed lines indicate r values over 0.5 or under -0.5. Black dotted lines indicate cutting point of correlation. B. Gene overrepresentation analysis plot for positive correlated genes in all three areas studied according to fold change and false discovery rate (FDR). C. Top Gene

Ontology terms for overrepresentation analysis of categories with over 500 genes (top) and under 500 genes (bottom) organized by number of genes and FDR values.

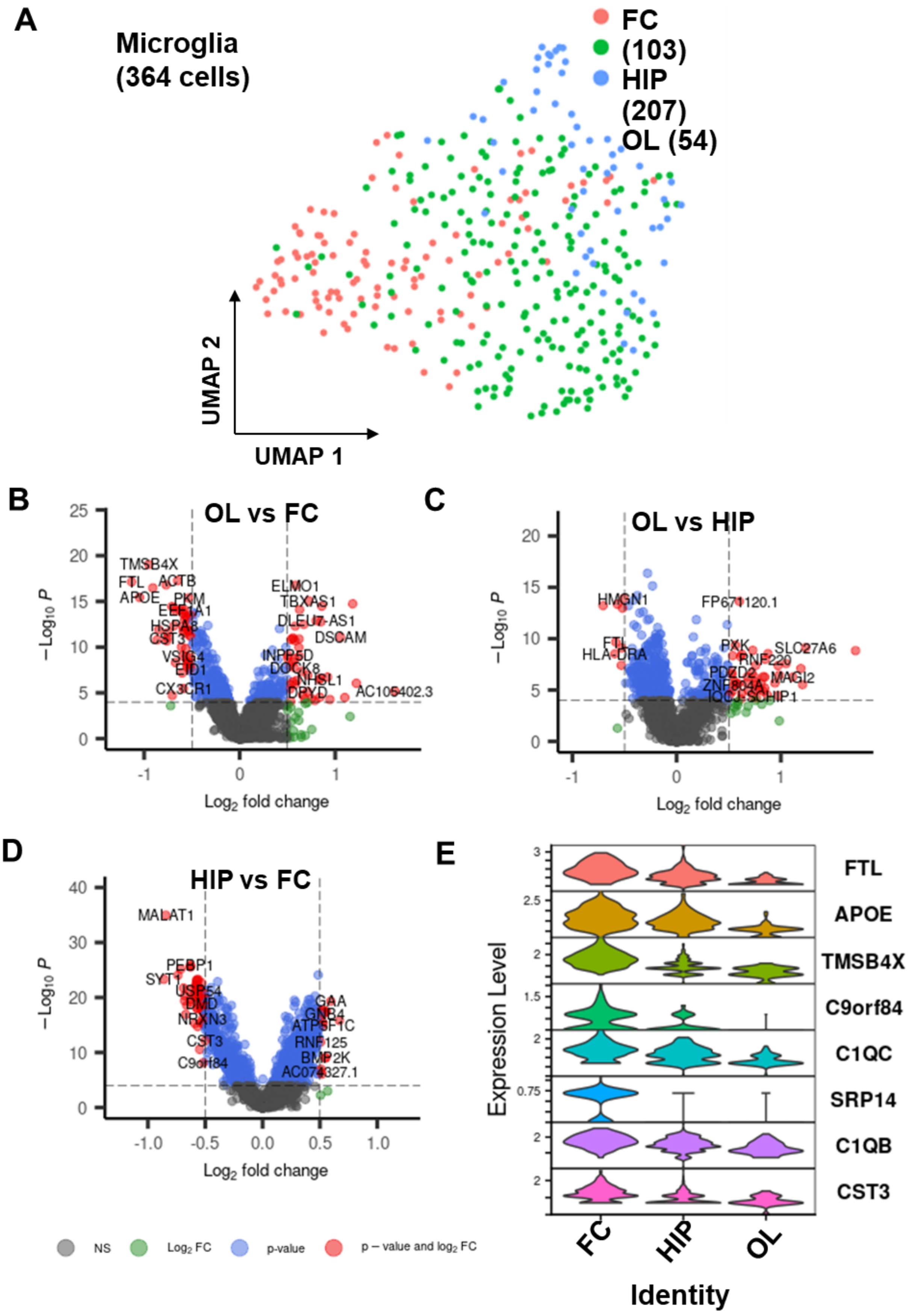
A
Microglia (364 cells)
FC (103)
HIP (207)
OL (54)
UMAP 2
UMAP 1
B
OL vs FC
C
OL vs HIP
D
HIP vs FC
E
Log2 fold change
−Log10 P
NS
Log2 FC
p-value
p − value and log2 FC
Expression Level
FTL
APOE
TMSB4X
C9orf84
C1QC
SRP14
C1QB
CST3
FC
HIP
OL
Identity

Supp. Fig. 18: DGE in microglia from an ADAD *PSEN1*E280A APOE3ch homozygote case. A. Dimesionality reduction scatterplot for microglia differentiating between frontal cortex, hippocampus, and occipital cortex. B. Volcano plot for DGE analysis between microglia from the occipital versus frontal cortex. C. Volcano plot for DGE analysis between microglia from the occipital versus hippocampus. D. Volcano plot for DGE analysis between microglia from the hippocampus versus frontal cortex. E. Violin plots for top genes identified.

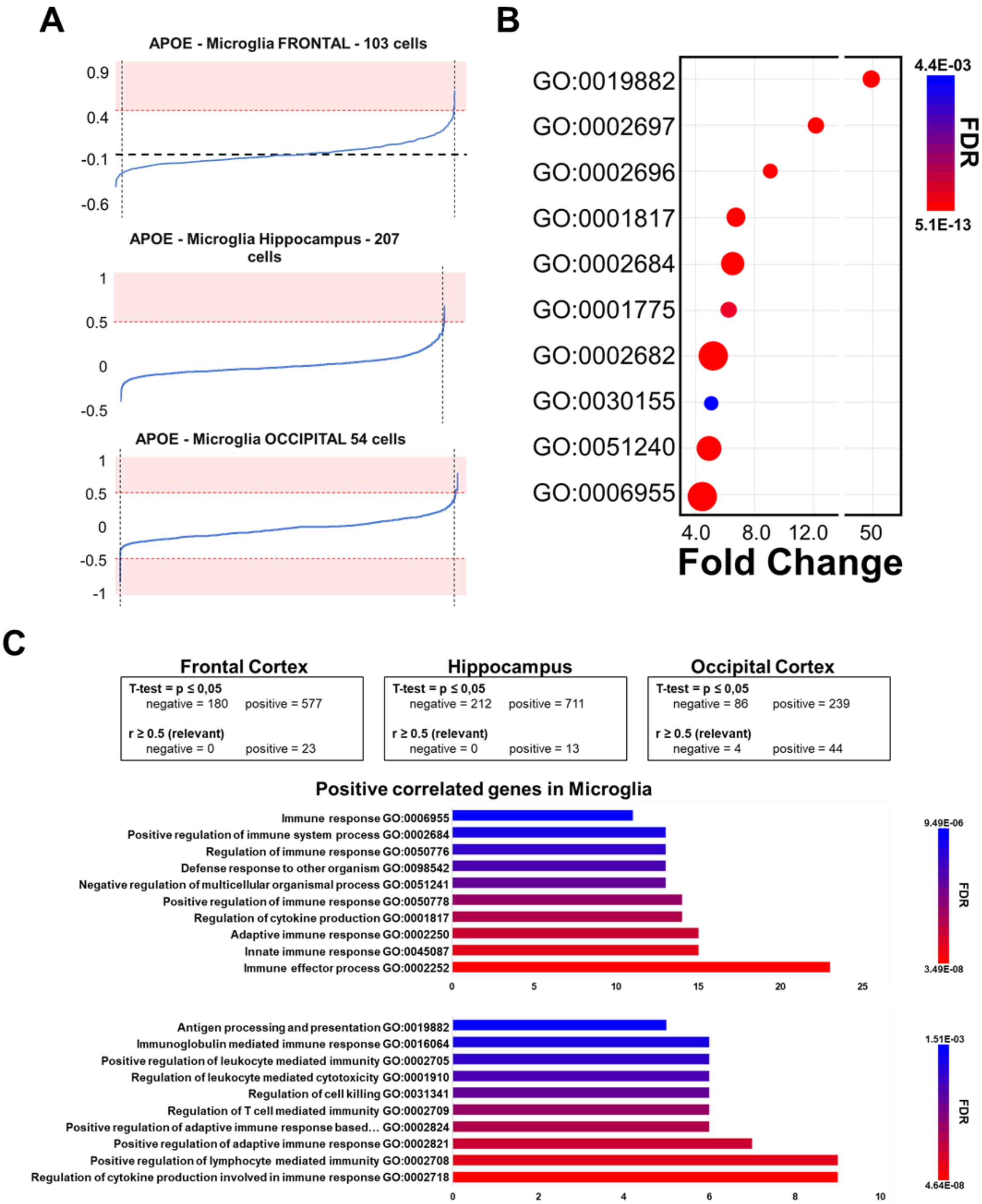

Supp. Fig. 19: Analysis of gene expression correlating with APOE expression in microglia of ADAD *PSEN1*E280A APOE3ch homozygote case. A. Correlation distribution of genes in frontal cortex, hippocampus and occipital cortex. Red dashed lines indicate r values over 0.5 or under -0.5. Black dotted lines indicate cutting point of correlation. B. Gene overrepresentation analysis plot for positive correlated genes in all three areas studied according to fold change and false discovery rate (FDR). C. Top Gene

Ontology terms for overrepresentation analysis of categories with over 500 genes (top) and under 500 genes (bottom) organized by number of genes and FDR values.

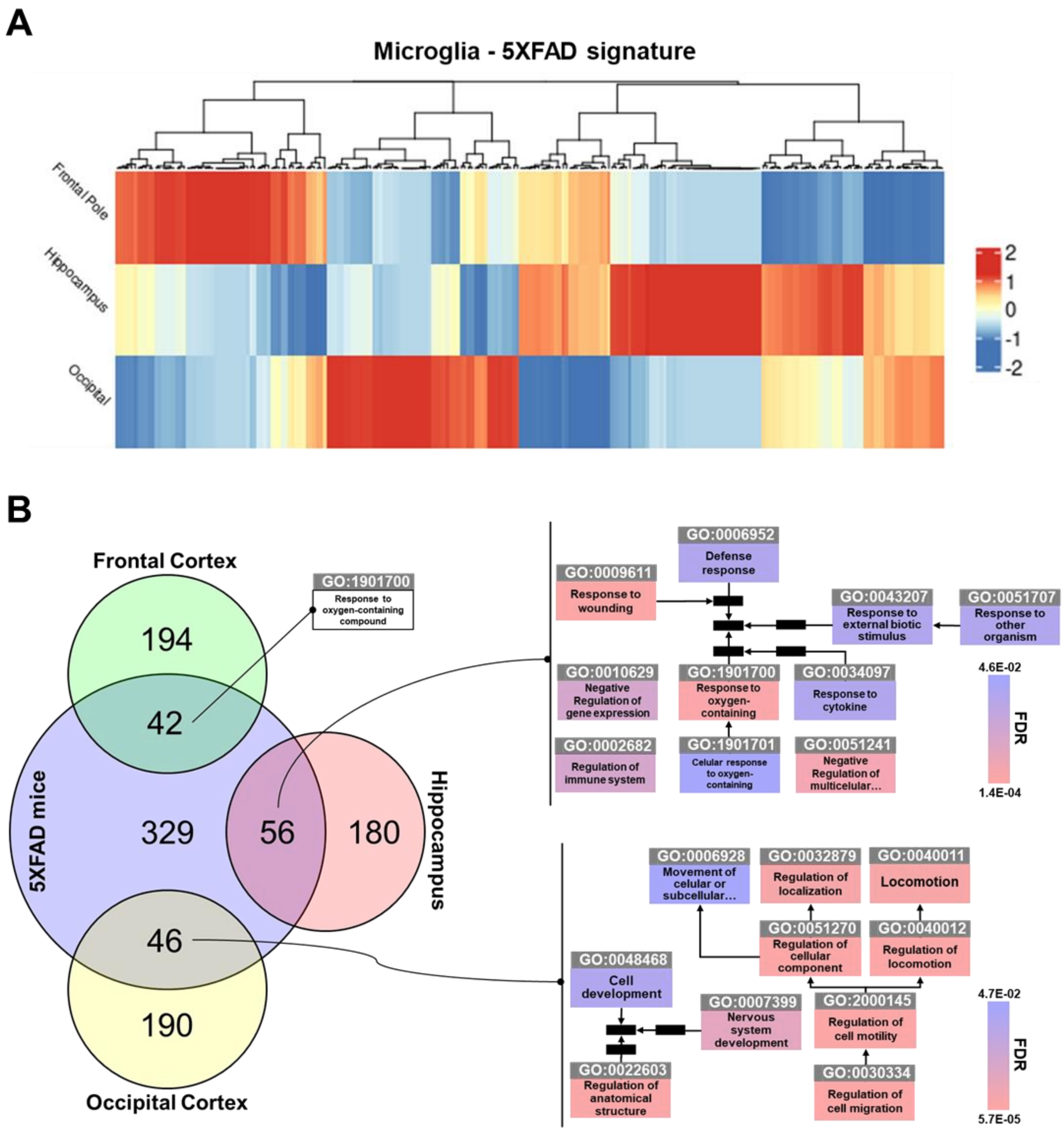

Supp. Fig. 20: Targeted DGE analysis for a chronic neurodegenerative signature according to Krasemann et al. 2017. A. Heatmap depicting top genes with differential expression profiles in microglia of an ADAD PSEN1 E280A APOEch homozygote case according to the genes identified for a chronic neurodegenerative profile in 5XFADtg mice. B. Top gene ontology terms as identified by overrepresentation analysis from the subset of genes exclusive to each area.

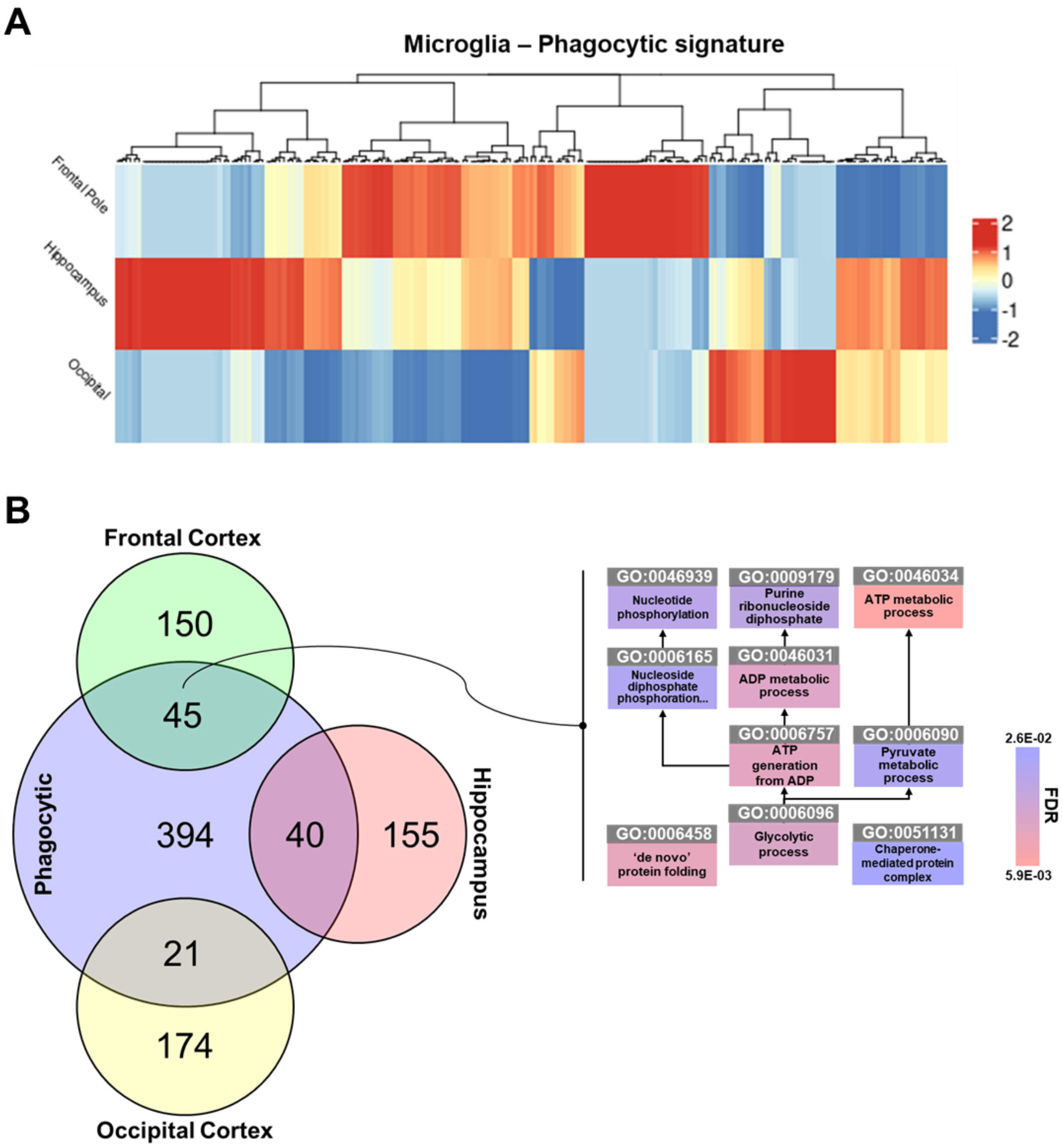

Supp. Fig. 21: Targeted DGE analysis for a phagocytic signature according to Krasemann et al. 2017. A. Heatmap depicting top genes with differential expression profiles in microglia of an ADAD PSEN1 E280A APOEch homozygote case according to the genes identified for a phagocytic profile in 5XFADtg mice. B. Top gene ontology terms as identified by overrepresentation analysis from the subset of genes exclusive to each area.

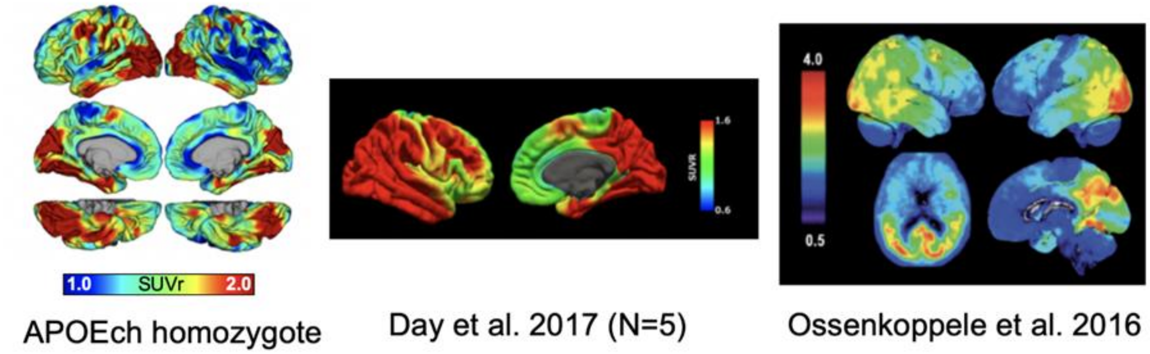

Supp. Fig. 22: Comparison of tau PET findings in the PSEN1E280A APOE3ch case to posterior cortical atrophy (PCA) cases from the literature. Even though the ADAD resistant case shows a predominant occipital pattern for tau pathology, it does not resemble tau pathology patterns as described by Day GS et al. 2017 (PMID: 28394771) and Ossenkoppele R et al. 2016 (PMID: 26962052).

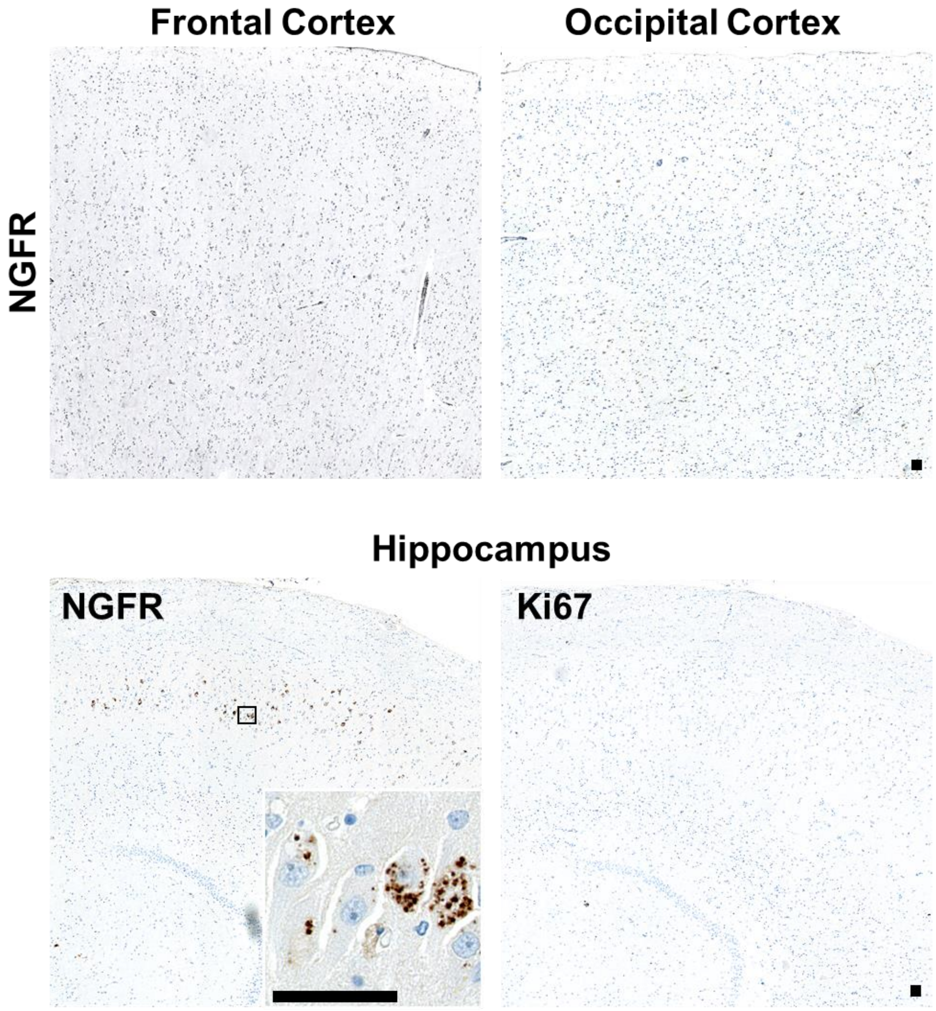

Supp. Fig 23: Screening for possible melanoma metastatic or proliferative cells in the frontal cortex, hippocampus, and occipital cortex from a *PSEN1*E280A APOE3ch homozygote case. An antibody against marker CD271 (Nerve Growth Factor receptor, NGFR) was used to identify melanoma cells. No metastatic cells were identified. Instead, NGFR was detected in pyramidal neurons of CA2 region in the hippocampus (inset). Likewise, no proliferation marker Ki67 signal was identified in the studied tissue. Bars = 50 μm.
